# Occult Neoplasms in Sudden Unexpected Death in Infancy: A Scoping Review of Post-Mortem Findings

**DOI:** 10.3390/diagnostics16193189

**Published:** 2026-09-30

**Authors:** Jessika Camatti, Maria Paola Bonasoni, Anita Nagy, Rossana Cecchi, Anna Laura Santunione, Erjon Radheshi

**Affiliations:** 1 Department of Medicine and Surgery, University of Parma, Via Università 12, 43121 Parma, Italy; jessika.camatti@unipr.it; 2 Pathology Unit, Azienda USL-IRCCS di Reggio Emilia, Via Amendola 2, 42122 Reggio Emilia, Italy; 3 Department of Laboratory Medicine & Pathobiology, University of Toronto, Toronto, ON M5S 1A1, Canada; anita.nagy@sickkids.ca; 4 Unit of Legal Medicine, Department of Biomedical, Metabolic and Neural Sciences, University of Modena and Reggio Emilia, Via Campi 287, 41125 Modena, Italy; rossana.cecchi@unimore.it (R.C.); annalaura.santunione@unimore.it (A.L.S.); 5 Unit of Legal Medicine and Bioethics, Azienda USL-IRCCS di Reggio Emilia, Via Amendola 2, 42122 Reggio Emilia, Italy; erjon.radheshi@ausl.re.it

**Keywords:** sudden unexpected death in infancy, SUDI, undiagnosed neoplasm, paediatric forensic autopsy, post-mortem pathology

## Abstract

**Background:** Sudden unexpected death in infancy (SUDI) may occasionally be attributable to neoplasms that were clinically unrecognised before the terminal event and diagnosed or confirmed during post-mortem investigation. These occult neoplasms are exceptionally rare and remain poorly characterised. This scoping review identifies published reports of neoplasms not diagnosed or strongly suspected before the terminal event in infants who died suddenly and unexpectedly, and systematically collects data on pathological features, diagnostic modalities, mechanisms of death, and the extent to which the neoplasm contributed to death. **Methods:** A search of PubMed/MEDLINE and Scopus was conducted without a lower publication-date restriction through 19 July 2026, in accordance with Joanna Briggs Institute guidance and PRISMA-ScR. Publications describing sudden unexpected deaths in infants younger than 12 months with a previously undiagnosed neoplasm were selected and reviewed. Findings were extracted, and deaths were classified according to whether the neoplasm was causal, contributory, or incidental to death. **Results:** Twenty-five studies comprising 32 infant cases were included in the review. The median age at death was 2.5 months. The most frequent lesions were cardiovascular or thoracic neoplasms (16/32), followed by haematological or lymphoid neoplasms (6/32). Sixteen lesions were benign, ten were malignant, and six were intermediate, uncertain, or tumour-like. Twenty-five neoplasms were evident on macroscopic examination, whereas seven were identified only histologically. Reported mechanisms of death included arrhythmia, mechanical obstruction, respiratory failure, haemodynamic compromise, haemorrhage, leukostasis, and diffuse infiltration. Within the published cases included in this review, the review team classified the neoplasm as causal in 16 cases (16/32, 50.0%), contributory in 13 cases (13/32, 40.6%), and incidental in three cases (3/32, 9.4%). **Conclusions:** Autopsy examination is critical for identifying previously undiagnosed neoplasms, which are rare but potentially important findings in the investigation of SUDI. Among the selected published cases included in this review, the review team classified the neoplasm as causal in half of cases; this proportion should not be extrapolated to the broader SUDI population. Comprehensive autopsy assessment of SUDI cases, including thorough histological sampling and ancillary testing, is therefore essential for determining the role of a tumour in infant death.

## 1. Introduction

Sudden unexpected death in infancy (SUDI) is an umbrella term encompassing deaths occurring suddenly and unexpectedly during the first year of life, regardless of whether a cause is ultimately identified. Its investigation represents a major challenge in paediatric pathology and forensic medicine because the terminal event is frequently unwitnessed, preceding symptoms may be absent or nonspecific, and pathological findings may be subtle or difficult to interpret. A reliable determination of the cause of death therefore requires a structured and multidisciplinary approach integrating the circumstances of death, death-scene investigation, clinical and family history, complete autopsy examination, and appropriately selected ancillary investigations [1].

SIDS remains a diagnosis of exclusion and should only be considered after a comprehensive post-mortem investigation. Systematic tissue sampling along with ancillary testing may reveal previously undiagnosed diseases and establish a cause of death even in the absence of significant macroscopic abnormalities, with the lungs and heart representing particularly important diagnostic targets [2]. Many autopsy studies have shown that explained SUDI encompasses a broad spectrum of infectious, cardiovascular, congenital, neurological, metabolic, and other natural diseases, and these conditions may have produced few or no recognisable symptoms during life [3].

The neonatal period, under the age of 28 days, presents additional diagnostic difficulties. During this period, there is physiological transition from intrauterine to extrauterine life, and sudden fatal deterioration may occur due to prematurity, birth asphyxia, infections, or congenital diseases. Clinicopathological studies of sudden unexpected neonatal deaths have demonstrated considerable heterogeneity in the underlying causes and have highlighted the importance of integrating detailed placental, neonatal, cardiac, pulmonary, microbiological, metabolic, and genetic investigations [4]. These observations reinforce the need to avoid premature classification of an infant death as unexplained when potentially identifiable pathological conditions have not been systematically excluded.

Increasing attention has consequently been directed towards “hidden” or under-recognised causes of SUDI. These include conditions that may remain clinically silent and may not be detectable through routine macroscopic examination alone, requiring targeted histological, microbiological, toxicological, biochemical, immunohistochemical, or molecular investigations. Post-mortem microbiology, in particular, can provide critical diagnostic information when infectious causes of death are not recognised during life, although its findings should be interpreted in conjunction with the clinical history, autopsy findings, histopathology, and appropriate sampling procedures [5]. A recent comprehensive review identified occult cardiac disorders, infections, inherited metabolic diseases, neurological abnormalities, toxicological exposures, environmental circumstances, and inflicted injury among the principal diagnostically challenging conditions within the SUDI spectrum [6]. Failure to recognise such conditions may result in misclassification as SIDS or unexplained death, with important consequences for mortality surveillance, family counselling, recurrence-risk assessment, and preventive interventions.

Neoplasms represent a further, exceptionally uncommon category of potentially hidden disease. In infancy, both benign and malignant lesions may remain undiagnosed because of their rarity, clinically silent evolution, or presentation with nonspecific manifestations. Although broad reviews of SUDI have extensively examined cardiac, infectious, metabolic, neurological, environmental, toxicological, and medico-legal conditions, recent organ-specific systematic investigations have further characterised infectious causes identified during post-mortem examination, including central nervous system infections [7]. Occult neoplasia, however, has not been specifically and systematically mapped in infants. The available evidence remains dispersed across individual case reports, small case series, and broader paediatric autopsy studies, frequently combining infants with older children and applying inconsistent criteria to distinguish directly causal, contributory, uncertain, and incidental neoplastic findings.

Therefore, the present scoping review aimed to identify and systematically map neoplasms not diagnosed or strongly suspected before the terminal event and identified or confirmed during post-mortem investigation in infants who died suddenly and unexpectedly. Specifically, the review sought to describe the demographic and clinical characteristics of the reported cases, the pathological spectrum and anatomical distribution of the neoplasms, the diagnostic methods employed, the proposed mechanisms of death, and the causal relationship between each lesion and the fatal event.

## 2. Materials and Methods

A scoping review was conducted according to the methodological guidance of the Joanna Briggs Institute and reported in accordance with the PRISMA extension for Scoping Reviews (PRISMA-ScR). The completed PRISMA-ScR checklist is provided as non-published material for editorial assessment. The protocol was developed before study selection and data extraction but was not prospectively registered.

The review aimed to identify neoplasms not diagnosed or strongly suspected before the terminal event and identified or confirmed during post-mortem investigation in infants who died suddenly and unexpectedly and to describe their pathological characteristics, diagnostic methods, proposed mechanisms of death, and causal relationship with the fatal event.

Eligible reports included live-born infants younger than 12 months who experienced a sudden and unexpected death or fatal collapse and in whom a neoplasm not diagnosed or strongly suspected before the onset of the terminal event was identified or confirmed through autopsy or another tissue-based post-mortem investigation. For the purposes of this review, SUDI was used in a broad descriptive sense encompassing sudden and unexpected death or fatal collapse in live-born infants younger than 12 months. Neonatal and perinatal presentations were retained when the neoplasm had not been diagnosed or strongly suspected before the terminal event; however, these cases were considered a distinct clinical subgroup from more typical post-neonatal SUDI presentations.

Benign, intermediate, and malignant solid tumours, haematological and lymphoid neoplasms, embryonal and germ-cell tumours, and tumour-like lesions conventionally classified among paediatric neoplasms were considered. Lesions considered as causal, contributory, uncertain, or incidental were all included.

Case reports, case series, letters containing sufficient original data, autopsy studies, pathology registries, and observational studies were eligible. Studies including broader paediatric populations were considered only when data concerning infants younger than 12 months could be extracted separately.

Foetal deaths, stillbirths, deaths occurring after 12 months of age, and cases in which the neoplasm had been diagnosed or strongly suspected before the onset of the terminal event were excluded. Congenital masses first recognised during the fatal perinatal event itself remained eligible. Deaths occurring during the expected clinical course or treatment of a known oncological or terminal disease were also excluded. Reports without pathological confirmation, conference abstracts lacking sufficient information, reviews, editorials, experimental studies, and animal studies were not included. Reviews were examined only to identify additional primary studies.

No publication-date or language restrictions were applied, provided that the report could be reliably interpreted.

PubMed/MEDLINE and Scopus were searched without a lower publication-date restriction through 19 July 2026. Five complementary searches were performed in each database, covering neoplasms independently of anatomical site and additional tumour categories involving the cardiovascular and thoracic systems, nervous system, haematological and lymphoid tissues, and visceral, endocrine, germ-cell, soft-tissue, vascular, skeletal, and cutaneous sites.

Terms relating to autopsy, post-mortem examination, necropsy, forensic investigation, and medicolegal death investigation were included in every search. The complete strategies and the number of records retrieved are reported in Supplementary File S1.

Titles and abstracts and, subsequently, full texts were independently assessed by two reviewers. Disagreements were resolved by consensus or consultation with a third reviewer. Reasons for full-text exclusion were recorded, and the selection process was summarised in a PRISMA flow diagram.

Data were independently extracted using a standardised form. Collected variables included publication characteristics, infant age and sex, medical history, preceding symptoms, circumstances of death, resuscitation and survival interval, tumour type, anatomical site, dimensions, histological diagnosis, gross visibility, ancillary investigations, proposed cause and mechanism of death, competing findings, and familial or genetic implications.

Multiple publications describing the same infant were treated as a single case, using the most complete report as the primary source.

The causal interpretation provided by the original authors was recorded separately from that of the review team. For the purposes of the review, neoplasms were classified using a three-tier framework informed by the WHO Medical Certificate of Cause of Death, which distinguishes conditions forming part of the causal sequence leading to death from other significant conditions contributing to death [8]. Neoplasms were therefore classified as (1) causal, when considered part of the causal sequence leading to death; (2) contributory, when they may have contributed to death but a direct causal role could not be established; or (3) incidental, when no convincing relationship with the fatal event was identified. As an operational rule for the present review, cases previously assessed as directly causal were included in the causal category, whereas those assessed as probably or possibly causal were included in the contributory category. The operational assessment considered five domains: anatomical compatibility between the lesion and the proposed fatal mechanism; severity and extent of organ involvement; direct or indirect evidence supporting the proposed physiological mechanism; completeness of the post-mortem investigation; and the presence of competing causes or contributory conditions. In the absence of sufficient evidence for inclusion in the direct causal sequence, but where a biologically plausible contribution remained, the lesion was classified as contributory. Incidental classification was reserved for lesions lacking a convincing anatomical or physiological relationship with the fatal event. This framework was qualitative and was not intended as a validated quantitative scoring system. Causal classification was independently performed by J.C. and M.P.B. Disagreements were resolved by discussion and consensus. No formal measure of inter-rater agreement was calculated, as the three-tier framework was used as a structured qualitative causal assessment rather than as a validated diagnostic scoring instrument.

Methodological quality was independently assessed by two reviewers using the Joanna Briggs Institute critical appraisal tools appropriate to study design: the Checklist for Case Reports for publications classified as case reports, the Checklist for Case Series for the case series, and the Checklist for Prevalence Studies for the retrospective autopsy studies. Disagreements were resolved by consensus, with consultation of a third reviewer when required. Critical appraisal was not used as an exclusion criterion but informed the interpretation of the findings.

Results were synthesised descriptively and narratively according to tumour category, anatomical site, diagnostic method, mechanism of death, and causal classification. Categorical variables were reported as frequencies and percentages, whereas continuous variables were summarised using appropriate measures of central tendency and dispersion. No meta-analysis was planned because of the expected clinical and methodological heterogeneity.

Illustrative archival pathology material: Representative gross, histological, and immunohistochemical images of selected neoplasms relevant to the spectrum identified in the review were obtained from archival paediatric pathology cases of A.N. These images were included solely to illustrate the morphological spectrum of the neoplastic entities discussed in the review. The archival illustrative cases were not included in the study-selection process, case-level dataset, descriptive analyses, or causal classification of the scoping review.

Use of generative artificial intelligence: During the preparation of the manuscript, ChatGPT (OpenAI, GPT-5.6 Sol) was used exclusively for English-language editing, grammatical correction, and stylistic refinement. The tool was not used for literature searching, study selection, data extraction, data analysis, causal classification, interpretation of the findings, or generation of scientific conclusions. All AI-assisted edits were reviewed and approved by the authors.

## 3. Results

### 3.1. Study Selection

**Figure 1:**
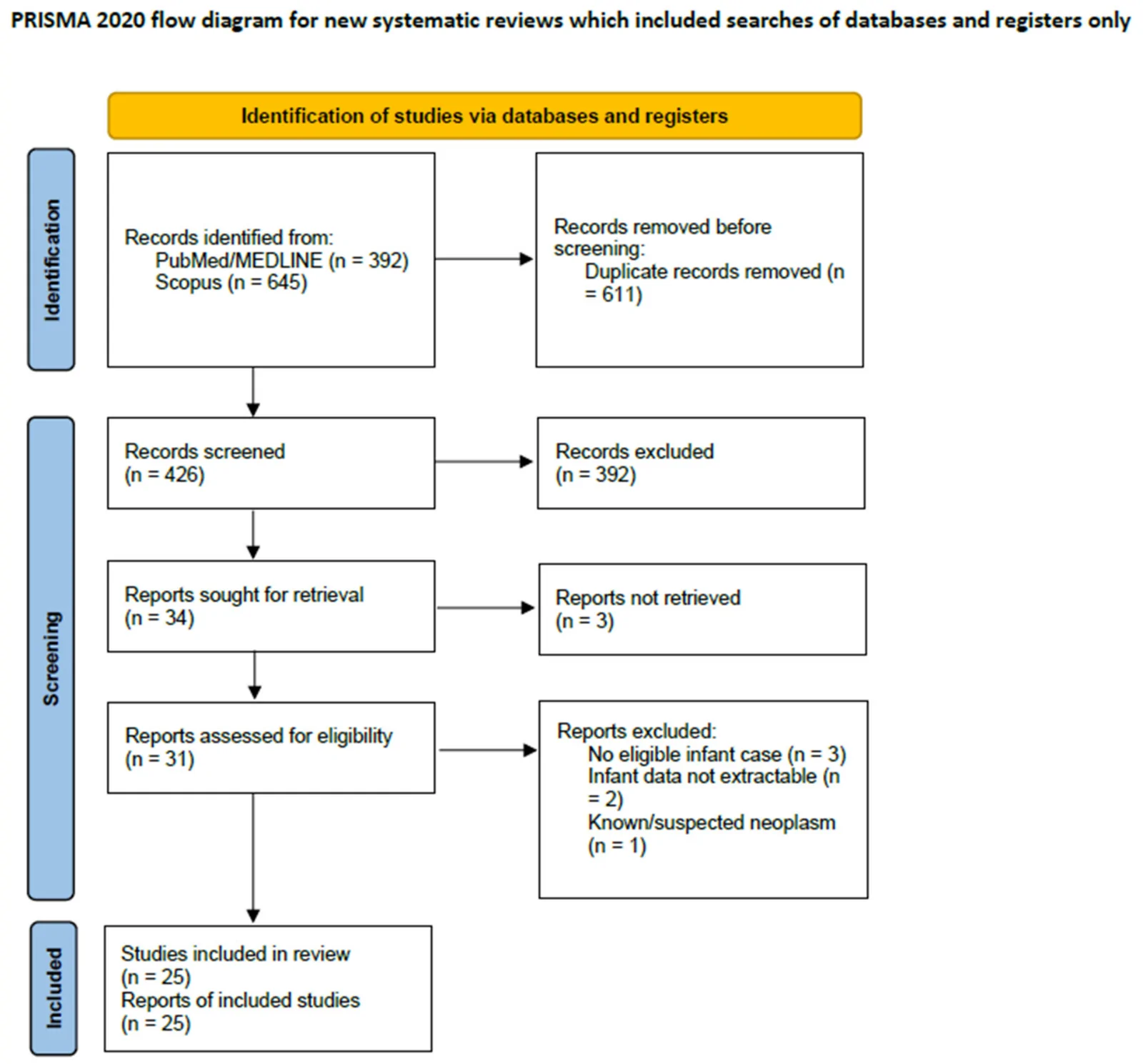
PRISMA 2020 flow diagram [34].

The database searches identified 1037 records, including 392 from PubMed/MEDLINE and 645 from Scopus. After removal of 611 duplicate records, 426 unique records underwent title and abstract screening. Of these, 392 were excluded because they did not meet the eligibility criteria, and 34 reports were sought for full-text retrieval. Three reports could not be retrieved, leaving 31 reports for full-text assessment. Six reports were subsequently excluded: three did not include an eligible infant case, two did not provide separately extractable data for infants younger than 12 months, and one concerned a neoplasm that had been diagnosed or strongly suspected before the onset of the terminal event. Twenty-five studies, represented by 25 reports, were therefore included in the review, providing data on 32 individual infant cases [9,10,11,12,13,14,15,16,17,18,19,20,21,22,23,24,25,26,27,28,29,30,31,32,33]. The study-selection process is presented in Figure 1.

### 3.2. Characteristics of the Included Evidence

The included studies were published between 1980 and 2026. The evidence base consisted predominantly of case reports, which accounted for 22 of the 25 studies. The remaining publications comprised one case series and two retrospective autopsy studies. Twenty-one studies contributed one eligible infant each, whereas four studies included multiple cases: four cases were extracted from Bryant et al., three from Byard et al., and two each from Defraia et al. and Somers et al.

The studies included medico-legal cases and next-of-kin consented autopsy examinations, and originated from forensic, coronial, paediatric pathology, and hospital autopsy settings across multiple geographical regions. Tissue-based pathological confirmation of the neoplasm was available in all included cases. The principal clinicopathological characteristics of the individual infants are presented in Table 1.

The complete case-level dataset, including clinical history, circumstances of death, gross and microscopic findings, ancillary investigations, competing findings, causal interpretation, and methodological appraisal, is provided in Supplementary Table S1.

### 3.3. Demographic and Clinical Characteristics

The 32 infants comprised 17 females and 15 males. Median age at death was 2.5 months, with a range from birth to 11 months. Six infants died during the neonatal period, 11 between 28 days and less than 3 months, 11 between 3 and less than 6 months, and four between 6 and less than 12 months.

No preceding symptoms or signs were reported in 15 cases (15/32, 46.9%). In the remaining 17 cases (17/32, 53.1%), the manifestations preceding death were generally nonspecific and included poor feeding, lethargy, irritability, vomiting, cough, dyspnoea, wheezing, fever, abdominal distension, bruising, bleeding, or an apparently isolated cardiac murmur.

In total, 21 cases (21/32, 65.6%) had a prior contact with healthcare services, and this included neonatal hospital care, routine paediatric or well-baby examinations, specialist follow-up, or medical assessment for symptoms initially attributed to common or apparently benign conditions. Despite these contacts, the underlying neoplasm remained undiagnosed before the fatal collapse.

Fifteen infants (15/32, 46.9%) were found unresponsive or dead, while a witnessed collapse or acute clinical deterioration was reported in 15 cases. Six events (6/32, 18.8%) occurred during feeding or within approximately one hour after feeding. Resuscitation was explicitly reported in 20 cases (20/32, 62.5%) and was unsuccessful in all cases for which the outcome was specified. These clinical and circumstantial categories were not mutually exclusive. The demographic and clinical distributions are summarised in Table 2.

### 3.4. Neoplasm Categories and Pathological Characteristics

Cardiovascular and thoracic neoplasms represented the largest diagnostic category, accounting for 16 of the 32 cases (50.0%). This group comprised 5 cardiac fibromas, 3 non-syndromic cardiac rhabdomyoma cases, 3 valvular haemangiomas, 1 primary cardiac sarcoma, 1 multifocal cardiac myxoma, 1 atrioventricular nodal neuroma-like lesion, 1 multifocal Purkinje-cell tumour or histiocytoid cardiomyopathy, and 1 pleuropulmonary blastoma.

Haematological and lymphoid neoplasms accounted for 6 cases (6/32, 18.8%). These comprised 3 cases of precursor B-cell acute lymphoblastic leukaemia, 2 cases of acute myeloid leukaemia, and 1 case of acute leukaemia for which a more specific lineage was not reported. These cases were generally characterised by widespread systemic, pulmonary, vascular, bone-marrow, or myocardial involvement.

The remaining 10 cases comprised 3 teratomas (intrapericardial, cervical, and sacrococcygeal), 3 hepatic or gastrointestinal tumours (2 infantile hepatic vascular tumours and 1 hepatoblastoma), 1 congenital adrenal neuroblastoma, 1 congenital mesoblastic nephroma, 1 thoracic extradural spinal lipoma, and 1 multisystem presentation of tuberous sclerosis complex associated with multiple cardiac rhabdomyomas and a subependymal giant-cell astrocytoma.

Sixteen neoplasms were classified as benign (16/32, 50.0%), 10 as malignant (10/32, 31.3%), and 6 as intermediate, uncertain, or tumour-like lesions (6/32, 18.8%). Multifocal or disseminated disease was present in 14 cases (14/32, 43.8%). The neoplasm was clearly visible at gross post-mortem examination in 25 cases (25/32, 78.1%), whereas 7 lesions were identified only microscopically (7/32, 21.9%). Five of the seven microscopic-only lesions involved the heart or cardiac conduction system, while the remaining 2 were haematological neoplasms characterised predominantly by microscopic vascular or visceral infiltration.

The largest tumour dimension was reported in 20 cases. Among these, the median maximum dimension was 42.5 mm, with a range from 0.75 to 120 mm. Marked variation was observed according to tumour type, ranging from minute valvular or conduction-system lesions to large cardiac, renal, hepatic, cervical, sacrococcygeal, and intrapericardial masses.

Representative gross, histological, and immunohistochemical appearances of selected neoplasms within the spectrum identified in the review are illustrated in Figure 2, Figure 3, Figure 4, Figure 5, Figure 6, Figure 7, Figure 8, Figure 9, Figure 10, Figure 11 and Figure 12 using archival cases provided by A.N. These illustrative cases were not included as additional cases in the scoping-review dataset.

### 3.5. Proposed Mechanisms of Death

The mechanisms by which tumours caused death were heterogeneous, and multiple mechanisms were frequently implicated. As more than one mechanism could be assigned to an individual case, these categories were not mutually exclusive.

An arrhythmia or cardiac conduction abnormality was considered in 14 cases (14/32, 43.8%). This mechanism was particularly relevant to cardiac fibromas, rhabdomyomas, Purkinje-cell tumours, and lesions located near the sinoatrial node, atrioventricular node, bundle of His, or bundle branches. Ventricular fibrillation was directly documented in one infant with a large septal cardiac fibroma. In most cases, however, the terminal rhythm was not documented, and the arrhythmic mechanism was proposed based on tumour location and associated anatomical changes.

Cardiac failure or haemodynamic compromise was proposed in 14 cases (14/32, 43.8%). Reported haemodynamic effects included obstruction of ventricular filling or outflow, compression of cardiac chambers, severe multivalvular involvement, restrictive myocardial infiltration, diffuse myocardial infiltration by leukaemic cells, and high-output failure caused by hepatic vascular shunting.

Respiratory failure or pulmonary involvement was implicated in 9 cases (9/32, 28.1%). The associated pathological findings included pleuropulmonary blastoma, pulmonary or intravascular blast infiltration, diffuse pulmonary leukostasis, pulmonary hypoplasia, and restriction of diaphragmatic excursion caused by marked hepatomegaly.

Mechanical obstruction or compression resulting in haemodynamic or respiratory failure was considered relevant in 9 cases (9/32, 28.1%). These included ventricular outflow-tract obstruction by cardiac fibromas, near-complete aortic and mitral valve obstruction by multifocal cardiac myxoma, compression of the heart and lungs by an intrapericardial teratoma, and severe tracheal compression and kinking caused by a congenital cervical teratoma.

Systemic haematological effects or diffuse neoplastic infiltration were identified in 6 cases (6/32, 18.8%). Reported effects included leukostasis, hyperviscosity, severe anaemia, marrow failure, tumour-lysis-associated hyperkalaemia, microvascular obstruction, and diffuse myocardial or multiorgan infiltration.

Haemorrhage, tumour rupture, or exsanguination was considered relevant in 4 cases (4/32, 12.5%). These included intra-abdominal haemorrhage from a congenital mesoblastic nephroma, traumatic rupture of a sacrococcygeal teratoma during delivery, haemorrhagic complications associated with acute leukaemia, and haemoperitoneum in an infant with hepatoblastoma.

Neurological or seizure-related mechanisms and infectious complications were each considered in 2 cases. In 5 cases, a specific tumour-related mechanism could not be demonstrated or remained substantially uncertain.

### 3.6. Causal Role of the Neoplasm

According to the assessment performed by the review team, the neoplasm was classified as causal in 16 cases (16/32, 50.0%), contributory in 13 cases (13/32, 40.6%), and incidental in three cases (3/32, 9.4%). In causal cases, the neoplasm was considered part of the causal sequence leading to death. Contributory cases involved a biologically plausible tumour-related role that could not be established as part of the causal sequence, or the presence of relevant competing pathological, clinical, or environmental factors. These included small or multifocal cardiac lesions without documented conduction-system involvement, hepatic vascular tumours without demonstrated cardiac failure, and neoplasms occurring in the presence of bronchopneumonia, major congenital cardiac abnormalities, or potentially hazardous sleeping conditions. In incidental cases, no convincing anatomical or physiological relationship between the lesion and death could be established, and either an alternative cause or a more plausible non-neoplastic explanation was present. The interpretation provided by the original authors and the independent three-tier causal assessment performed by the review team are reported separately in Supplementary Table S1.

### 3.7. Post-Mortem Diagnostic Investigations and Methodological Appraisal

Histological examination provided diagnostic confirmation in all included cases. Immunohistochemistry was reported in 11 cases (11/32, 34.4%) and was used principally to characterise haematological neoplasms, cardiac sarcoma, rhabdomyomas, cardiac myxoma, hepatic vascular tumours, and other diagnostically challenging lesions.

Molecular, cytogenetic, or targeted genetic investigations were reported in three cases (3/32, 9.4%). These comprised post-mortem testing for Finnish long-QT-syndrome founder variants, which was negative; cytogenetic and molecular confirmation of a KMT2A-MLLT1 rearrangement in infantile B-cell acute lymphoblastic leukaemia; and molecular analysis in an infant with precursor B-cell acute lymphoblastic leukaemia included in a retrospective autopsy series, for which case-specific molecular results were not reported in detail.

Post-mortem imaging was explicitly described in only two cases (2/32, 6.3%).

The methodological appraisal indicated that case identification and pathological confirmation were generally adequately reported. Several publications provided detailed clinical histories, death-scene information, complete gross and microscopic examinations, immunohistochemistry, or specialised serial examination of the cardiac conduction system. Reporting completeness nevertheless varied substantially, which may reflect limited clinical information available at the time of autopsy examination, as well as the absence of gross findings to prompt additional testing or more extensive histological sampling by the pathologist. Recurrent limitations included incomplete descriptions of the circumstances of death, limited toxicological, microbiological and molecular investigation, no examination of the cardiac conduction system and absence of ECG when an arrhythmic mechanism was proposed. The retrospective autopsy studies provided defined study periods and denominators but comparatively limited case-level information. Methodological quality was not used as an exclusion criterion.

## 4. Discussion

This scoping review provides an infant-specific synthesis of occult neoplasms identified during the post-mortem investigation of sudden unexpected death. The available evidence does not allow estimation of their prevalence, because it is predominantly derived from case reports and selected autopsy series. These findings suggest that the fatal potential of neoplasms in SUDI is more closely related to anatomical location, functional effects, haemodynamic disturbance, or electrical instability than to tumour biology alone. Malignancy was therefore not a prerequisite for lethality. Several histologically benign or tumour-like lesions were capable of disrupting vital functions through mechanical compression or obstruction, particularly when arising in anatomically constrained sites such as the heart, including the cardiac valves, ventricular outflow tracts, and pericardial cavity, as well as the upper airway [11,13,15,16,18,19,20,21,23,24,26,28,29,31]. Conversely, a malignant tumour may remain clinically occult until diffuse infiltration, haemorrhage, leukostasis, or metabolic decompensation occurs [9,10,12,27,30,32,33]. Thus, conventional distinctions between benign and malignant neoplasms are insufficient for post-mortem causal interpretation and should be interpreted in the context of other findings and results.

This principle is particularly relevant to cardiovascular neoplasms. Large cardiac masses may provide a convincing anatomical explanation when they substantially compromise ventricular filling, obstruct an outflow tract, deform a valve, or compress a cardiac chamber [11,14,20,23,28]. Greater interpretative difficulty arises with small or microscopic lesions situated near the conduction system. Various small lesions in the myocardium may plausibly create an arrhythmogenic substrate despite their limited volume [18,22,25,26,29]. However, anatomical proximity to the atrioventricular node, bundle of His, or bundle branches does not by itself prove that a lethal arrhythmia occurred.

Terminal electrocardiographic information was generally unavailable, and electrical mechanisms were therefore frequently inferred from tumour location, myocardial compression, or presumed involvement of conduction pathways [11,18,20,23,25,26,29]. The case reported by Ottaviani et al., in which ventricular fibrillation was documented in association with a large septal cardiac fibroma, offers unusually direct support for an arrhythmic interpretation [28]. In most other cases, however, the wording of the cause of death should remain appropriately qualified. A cardiac lesion should not automatically be equated with a fatal arrhythmia without consideration of the clinical history, circumstances of death, resuscitation findings, competing abnormalities, and completeness of the cardiac examination. When a sudden arrhythmogenic death is suspected, detailed myocardial sampling including examination of the conduction system, molecular testing and toxicological screening may substantially strengthen or weaken the proposed causal relationship [1,2,6].

Ante-mortem diagnosis of neoplasm was often difficult because affected infants were either apparently well or presented with non-specific manifestations commonly encountered in paediatric practice [9,10,11,12,16,19,21,24,27,30,32,33]. Minor respiratory symptoms, feeding difficulties, irritability, fever, or vomiting have low specificity in isolation. Nevertheless, persistent or progressive abdominal enlargement, a prominent or newly detected cardiac murmur, unexplained bruising or bleeding, organomegaly, respiratory deterioration, or marked haematological abnormalities may warrant further investigation, particularly when multiple findings coexist [10,12,23,27,32,33]. Congenital neoplasms may also present during the neonatal period as respiratory compromise, circulatory collapse, metabolic disturbance, or presumed sepsis [12,13].

The mechanisms by which tumours caused death were heterogeneous, and multiple mechanisms were frequently implicated. Cardiac masses may simultaneously impair filling, obstruct blood flow, reduce cardiac output, and produce electrical instability [11,14,20,23,28]. Congenital teratomas may compromise both ventilation and circulation through compression of the heart, lungs, or upper airway, while traumatic rupture may result in catastrophic haemorrhage [13]. Hepatic vascular tumours may cause high-output failure through abnormal vascular shunting and concurrently restrict diaphragmatic excursion because of hepatomegaly [16,24]. Acute leukaemias may produce severe anaemia, marrow failure, leukostasis, hyperviscosity, pulmonary microvascular obstruction, electrolyte disturbance, and myocardial or multiorgan infiltration [10,12,27,32,33]. In these situations, death may represent the convergence of several physiological disturbances rather than the consequence of one isolated mechanism.

This interaction becomes particularly important when neoplastic findings coexist with infection, congenital abnormalities, or potentially hazardous sleep circumstances [15,19,21,24,30,31]. The lesion may have been the primary cause of death, a contributory vulnerability that reduced the infant’s ability to withstand an additional stressor, or an incidental finding unrelated to death. Such cases are consistent with the broader understanding of SUDI as a heterogeneous event in which intrinsic vulnerability and external or superimposed stressors may coexist [1,3,4,6]. They also illustrate why a strictly monocausal interpretation may be inappropriate when several plausible contributors are present.

A central contribution of this review is therefore the explicit separation of lesion detection from causal attribution. Rare or visually striking abnormalities may acquire disproportionate explanatory weight at autopsy, particularly when no other immediate cause is identified. However, the presence of a neoplasm does not establish that it caused death. Causality is most convincing when the lesion demonstrably obstructs or compresses a vital structure, produces major haemorrhage, extensively infiltrates a critical organ, or is associated with a documented physiological disturbance [10,13,14,20,23,27,28,32,33]. Cautious interpretation of the findings is advised when the lesion is minute, or when a sufficient competing cause is present. Minute valvular lesions without obstruction, microscopic proliferations adjacent to structurally preserved conduction tissue, and a spinal lipoma without demonstrable cord or respiratory-pathway compression illustrate the limitations of attributing death solely on the basis of anatomical coexistence [15,22,31].

The WHO-informed three-tier framework adopted in this review allows causal attribution to distinguish lesions forming part of the causal sequence leading to death from conditions that may have contributed to death without sufficient evidence for their inclusion in that sequence. Accordingly, cases in which a tumour-related mechanism remained probable or possible were classified as contributory. This approach preserves biological plausibility while avoiding over-attribution of an incompletely demonstrated mechanism as the cause of death. The distinction is particularly relevant to presumed arrhythmic deaths without rhythm documentation, multifocal cardiac lesions without demonstrated involvement of conduction tissue, and vascular tumours in which the expected haemodynamic consequences were not established [15,16,18,21,24,25,26,29,30]. Conversely, classification as incidental was reserved for lesions without a convincing anatomical or physiological relationship to the fatal event. A structured causal assessment should therefore integrate anatomical compatibility, severity of organ involvement, evidence supporting the proposed mechanism, completeness of the post-mortem investigation, and the presence of alternative explanations.

The findings also have direct implications for autopsy practice. A macroscopically normal heart or apparently unremarkable viscera cannot exclude clinically significant neoplastic disease. Systematic histological sampling remains essential, together with targeted examination when organomegaly, lymphadenopathy, marrow pallor, unexplained haemorrhage, focal myocardial alteration, or evidence of systemic infiltration is present [1,2,6]. Particular attention should be directed to the heart and conduction system when the circumstances suggest sudden electrical collapse or when even a subtle septal, valvular, or subendocardial abnormality is identified.

Ancillary investigations should be guided by the pathological scenario. Immunohistochemistry helps to establish the diagnosis of haematological neoplasms, cardiac sarcomas, rhabdomyomas, vascular tumours, and other diagnostically challenging lesions [10,14,18,20,23,24,26,27,32,33]. The limited use of molecular testing and post-mortem imaging within the available literature probably reflects the historical period of many reports rather than a lack of current diagnostic value. Ancillary investigations include, but are not limited to, immunophenotyping, flow cytometry, cytogenetics, next-generation sequencing (NGS), other molecular studies, and imaging. Therefore, obtaining appropriate samples, such as fresh, frozen, or formalin-fixed tissue, for additional testing is critical at the time of autopsy examination.

Post-mortem diagnosis may also have consequences beyond certification of the individual death. Although many infantile neoplasms are sporadic, multiplicity of lesions, unusual tumour combinations, or associated developmental abnormalities may indicate a syndromic condition. The coexistence of multiple rhabdomyomas and tuberous sclerosis is a clear example [21]. Molecular characterisation of infantile leukaemia may also refine disease classification and inform family counselling, although it does not necessarily imply inherited susceptibility [27]. Referral for specialist paediatric or clinical genetic assessment should therefore be considered when the pathological pattern, family history, or molecular findings raise concern.

The available evidence has important limitations. Publication bias is likely to favour rare, anatomically striking, and apparently causal lesions, while incidental or equivocal findings may remain unpublished. The results cannot therefore be used to determine the incidence of occult neoplasia in SUDI or to compare the true frequency of different tumour categories. Accordingly, all frequencies reported in this review should be interpreted as descriptive proportions within the published case literature and not as estimates of population prevalence, incidence, or relative tumour frequency. Reporting quality was heterogeneous, particularly regarding death-scene investigation, terminal rhythm, resuscitation findings, toxicology, microbiology, molecular testing, and examination of the conduction system. These omissions limited the reconstruction of physiological mechanisms and reduced certainty in the causal classification. Importantly, absence of documentation was treated as missing information and was not interpreted as evidence that a specific investigation had been performed with negative results.

The literature also spans several decades during which paediatric tumour taxonomy and the classification of vascular, hamartomatous, and developmental lesions changed substantially, as well as the available diagnostic techniques. Some entities historically described as neoplasms may now be classified as hamartomatous or tumour-like proliferations. Their inclusion was appropriate to the scope of this review because they were reported within the differential diagnosis of neoplastic disease and could potentially interfere with vital functions, but it inevitably increased pathological heterogeneity. Historical diagnoses were therefore interpreted in their original diagnostic context, and no attempt was made to retrospectively impose contemporary classifications when the information reported in the original publication was insufficient to support formal reclassification.

The standardised causal assessment undertaken by the review team represents both a strength and a limitation. It allowed consistent criteria to be applied across publications in which causal language was variable or undefined. Nevertheless, the assessment remained retrospective and dependent on the completeness of the reported information. Arrhythmic, haemodynamic, respiratory, and metabolic mechanisms cannot be definitively reconstructed in the absence of full clinical history.

## 5. Conclusions

Occult neoplasms are an uncommon but important consideration in the investigation of SUDI. Their fatal potential depends primarily on anatomical location and functional consequences rather than on biological behaviour alone. Comprehensive autopsy, systematic histological sampling, targeted examination of the heart and conduction system, and appropriately selected ancillary investigations are essential for detection. Causal attribution should remain structured and cautious, distinguishing neoplasms forming part of the causal sequence leading to death from contributory conditions and incidental findings, while considering competing explanations. Standardised reporting and multicentre studies integrating histological, immunophenotypic, cytogenetic, molecular, and imaging methods are needed to improve recognition and interpretation of these rare lesions.

## Figures and Tables

**Figure 2:**
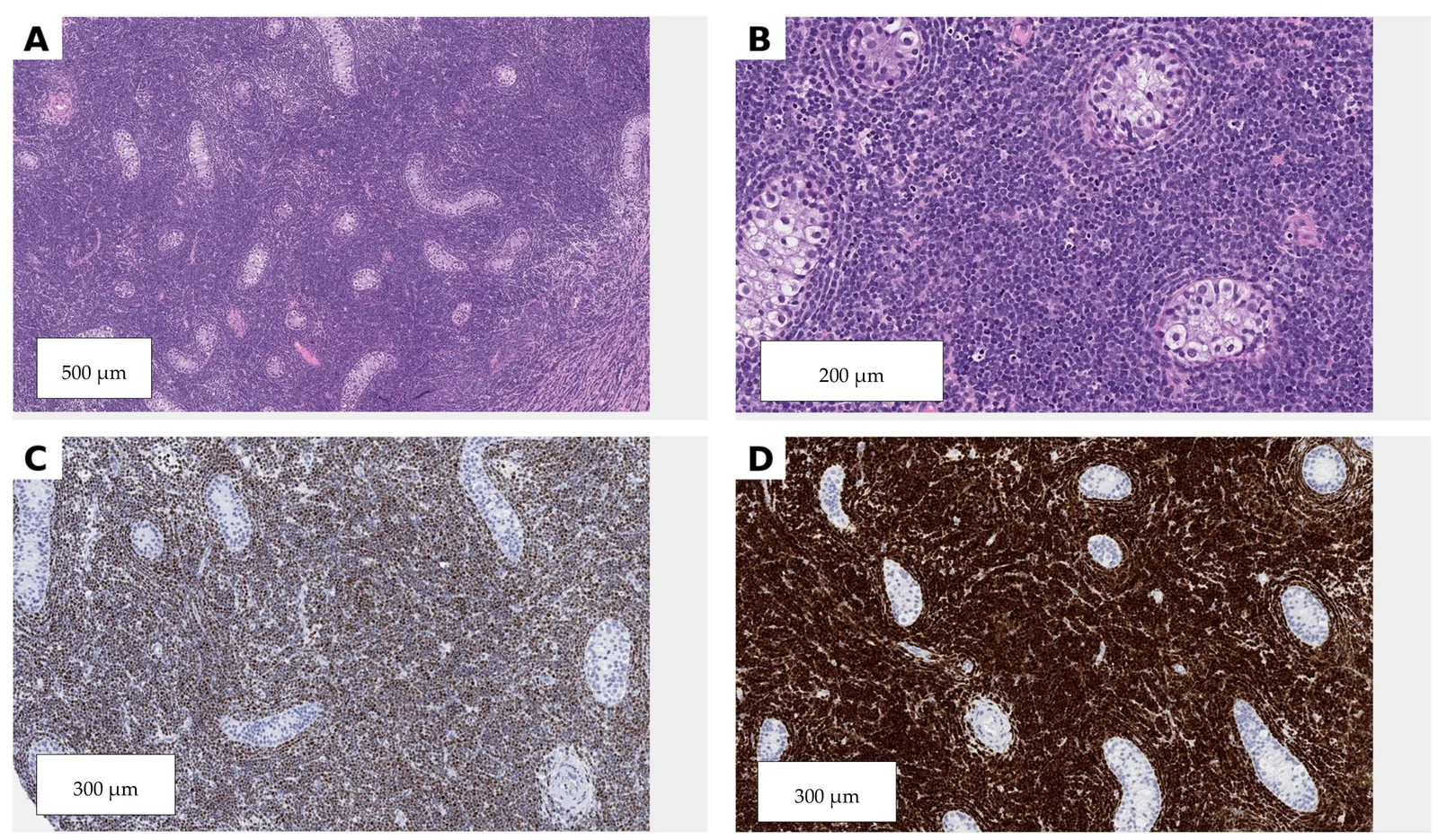
Representative archival histological and immunohistochemical appearances of **precursor B-cell acute lymphoblastic leukaemia infiltrating the testis** from a case of A.N. (**A**,**B**) H&E, ×5 and ×20, respectively. The images show diffuse interstitial infiltrate of uniform lymphoblasts separating and compressing seminiferous tubules. Lymphoblasts are PAX5 (**C**) and TdT nuclear positivity (**D**), respectively, ×10. This archival case is presented for illustrative purposes only and was not included as an additional case in the scoping-review dataset.

**Figure 3:**
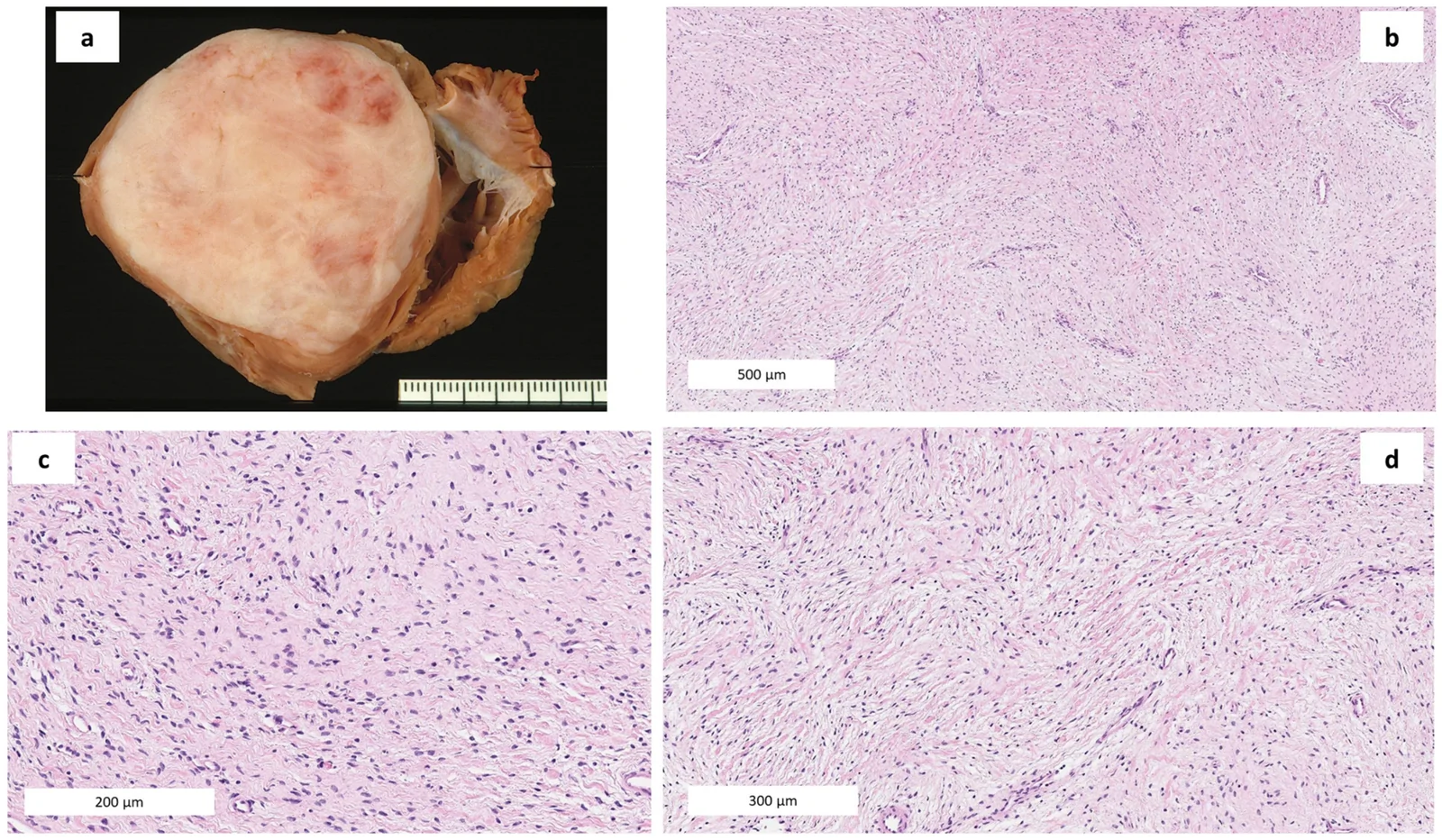
Representative archival gross and histological appearances of **cardiac fibroma** from a case of A.N. Gross appearance of a cardiac fibroma involving the interventricular septum (**a**). The lesion is typically solitary, firm, well-circumscribed, often unencapsulated, with a whorled, fibroid-like, gray-white cut surface. Histologically, cardiac fibroma is composed of bland monomorphic spindle cells (fibroblasts/myofibroblasts) in abundant dense collagenous stroma (**b**–**d**; H&E, ×5, ×20, and ×10, respectively). This archival case is presented for illustrative purposes only and was not included as an additional case in the scoping-review dataset.

**Figure 4:**
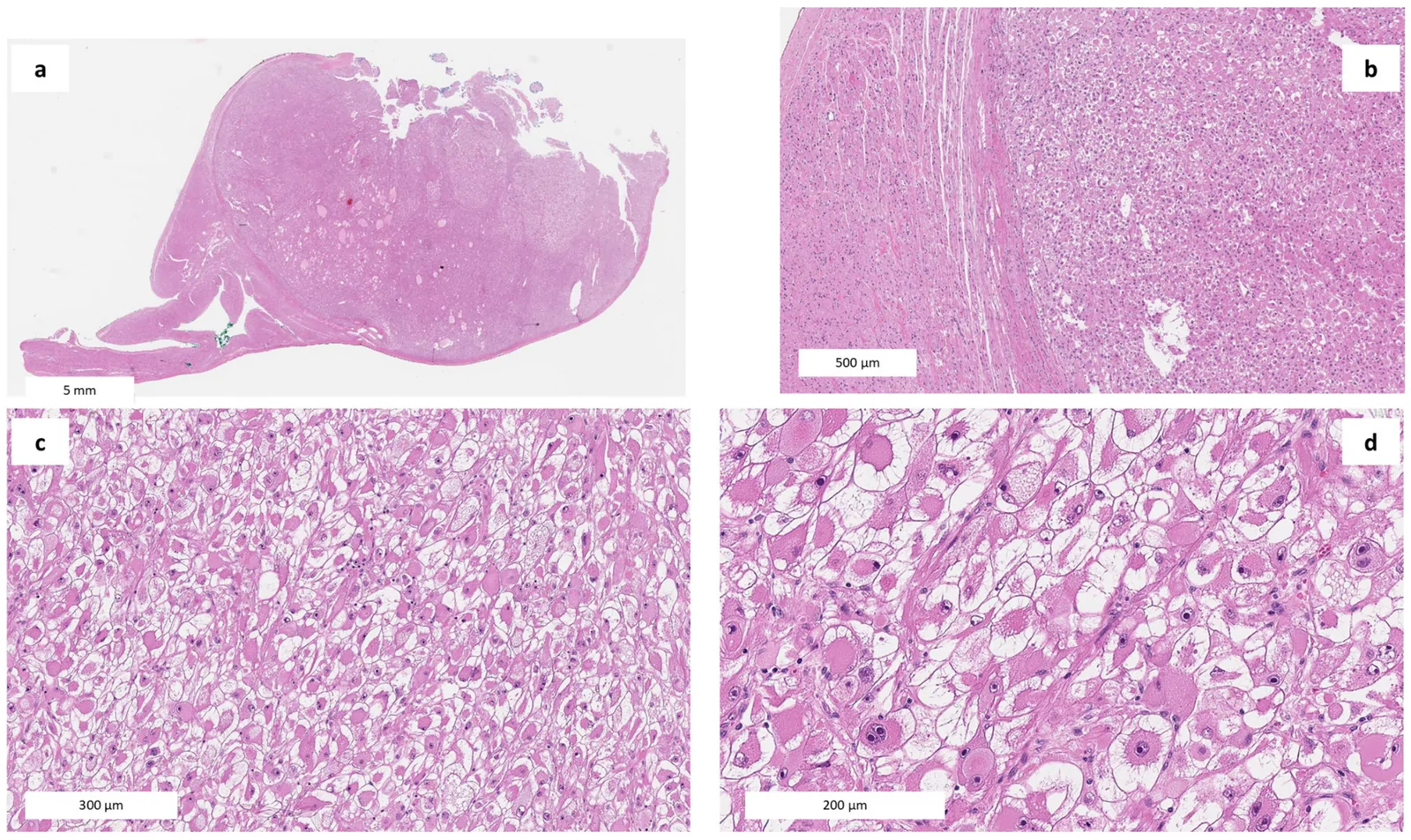
Representative archival histological appearances of **cardiac rhabdomyoma involving the right atrium** from a case of A.N. The tumor usually appears as a well-circumscribed, lobulated, expansile nodular growth at low power; with no infiltrative border (**a**,**b**, H&E, ×0.4, ×4). Cardiac rhabdomyoma is histologically characterized by nodules of markedly enlarged, rounded-to-polygonal cardiomyocytes with abundant, pale, vacuolated cytoplasm due to massive intracytoplasmic glycogen accumulation (**c**,**d**, H&E, ×10, and ×20, respectively). The hallmark cell is the “spider cell”, which consists of a central nucleus (or occasionally peripheral) suspended by thin, radiating cytoplasmic strands (myofibrillar processes) extending from the perinuclear zone to the cell membrane across a clear glycogen-filled cytoplasmic lake. The strands create the spider-in-web appearance at low-to-intermediate power (**c**, H&E, ×10). This archival case is presented for illustrative purposes only and was not included as an additional case in the scoping-review dataset.

**Figure 5:**
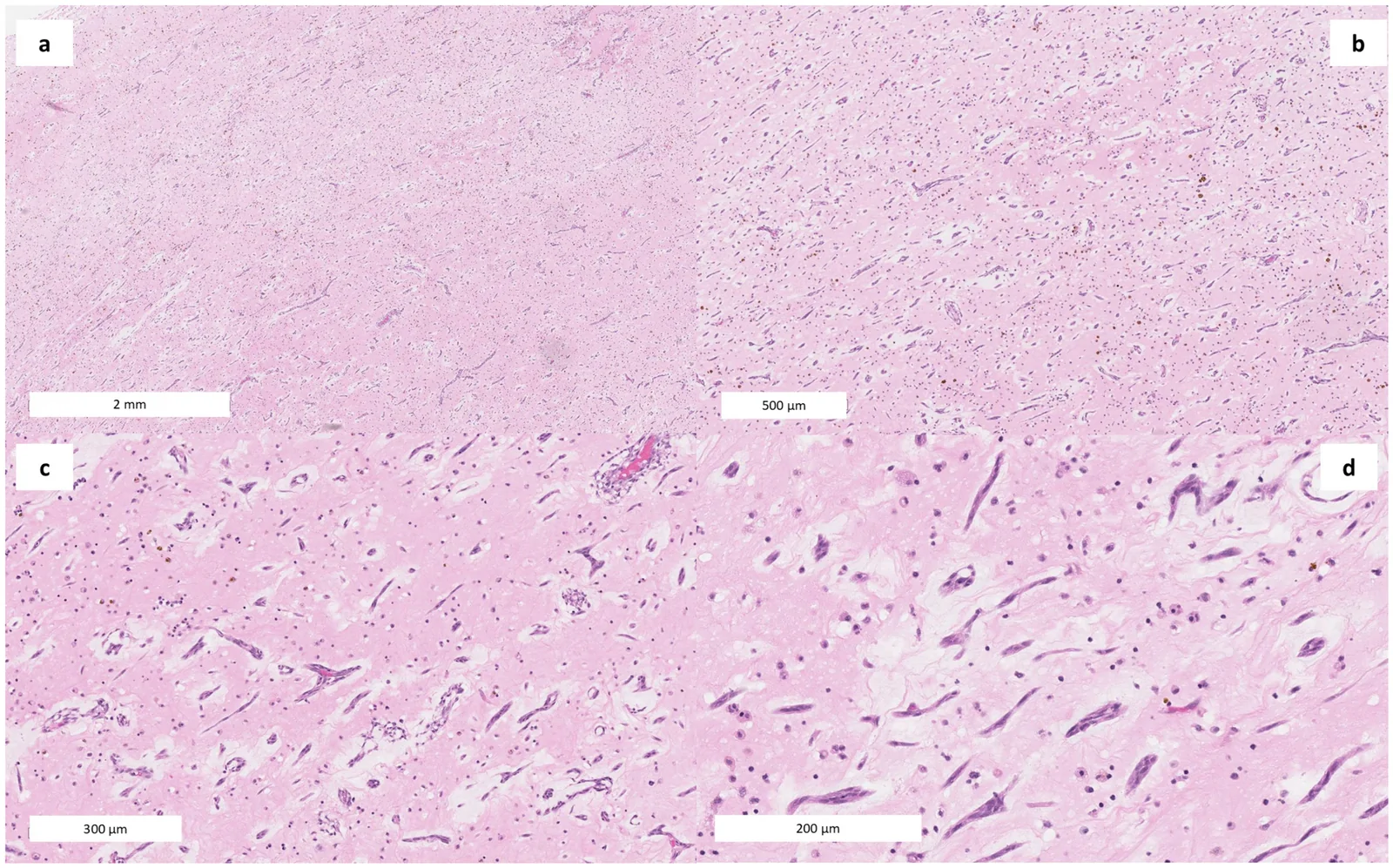
Representative archival histological appearances of **cardiac myxoma** from cases of A.N. In these images, the cardiac myxoma shows bland spindle myxoma cells with focal perivascular “syncytial cuffs (**c**, upper right) within an abundant basophilic myxoid (mucopolysaccharide-rich) stroma, often with delicate thin-walled vessels (**a**–**d**; H&E, ×2, ×4, ×10, and ×20, respectively).

**Figure 6:**
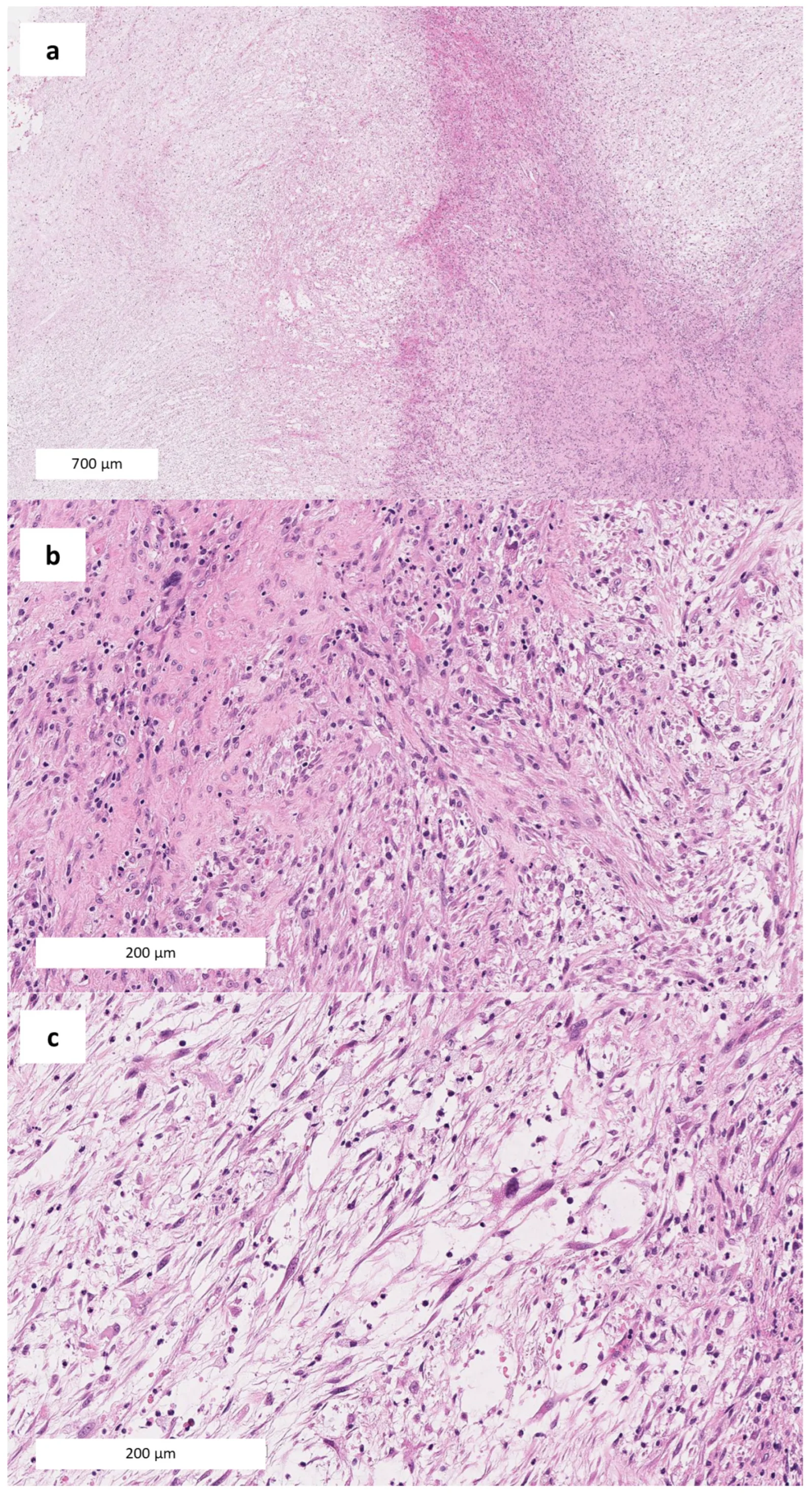
(**a**–**c**): Representative archival histological appearances of **unclassified cardiac sarcoma** from cases of A.N. Unclassified (undifferentiated) cardiac sarcoma is a high-grade malignant mesenchymal tumor lacking recognizable lineage-specific differentiation (angiosarcomatous, rhabdomyoblastic, or synovial), representing by definition a diagnosis of exclusion after thorough morphological, immunohistochemical, and molecular workup. The pictures show diffuse sheets of markedly pleomorphic spindle-to-epithelioid cells with hyperchromatic irregular nuclei, and prominent nucleoli, within a variable myxoid/fibrous stroma (H&E, ×2, ×20, ×20, respectively). These archival cases are presented for illustrative purposes only and were not included as additional cases in the scoping-review dataset.

**Figure 7:**
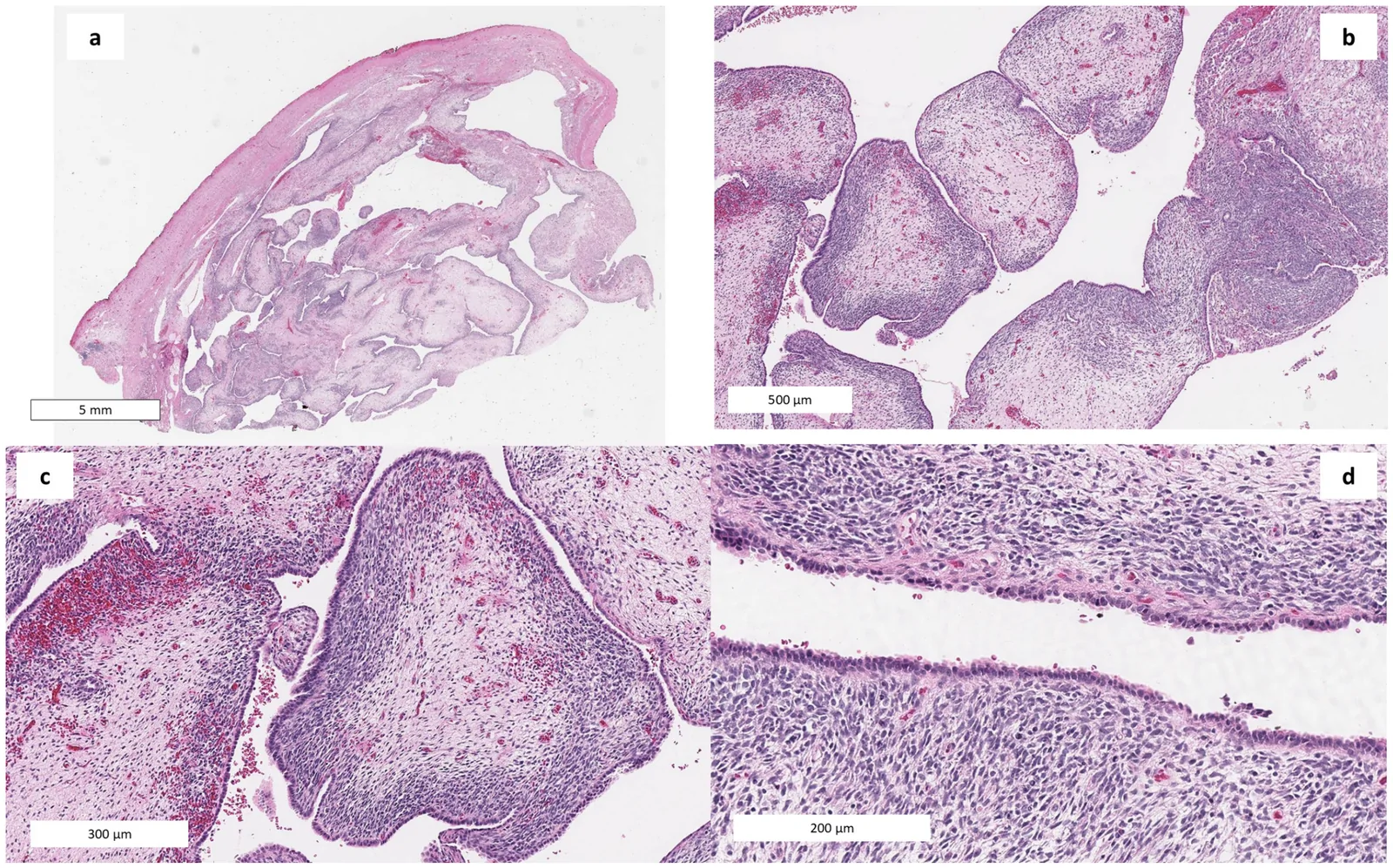
Representative archival histopathological appearances of **cystic pleuropulmonary blastoma (PPB type I)** from cases of A.N. (**a**–**d**): The lesion is composed of a multilocular cyst (**a**–**c**) lined by benign flat-to-low cuboidal epithelium with a subepithelial cambium layer-like concentration of primitive small round-to-spindle neoplastic mesenchymal cells (**d**) (H&E, shown at ×0.5, ×4, ×10, and ×20, respectively). Archival cases provided by A.N.; these images are presented for illustrative purposes and do not represent additional cases included in the scoping-review dataset.

**Figure 8:**
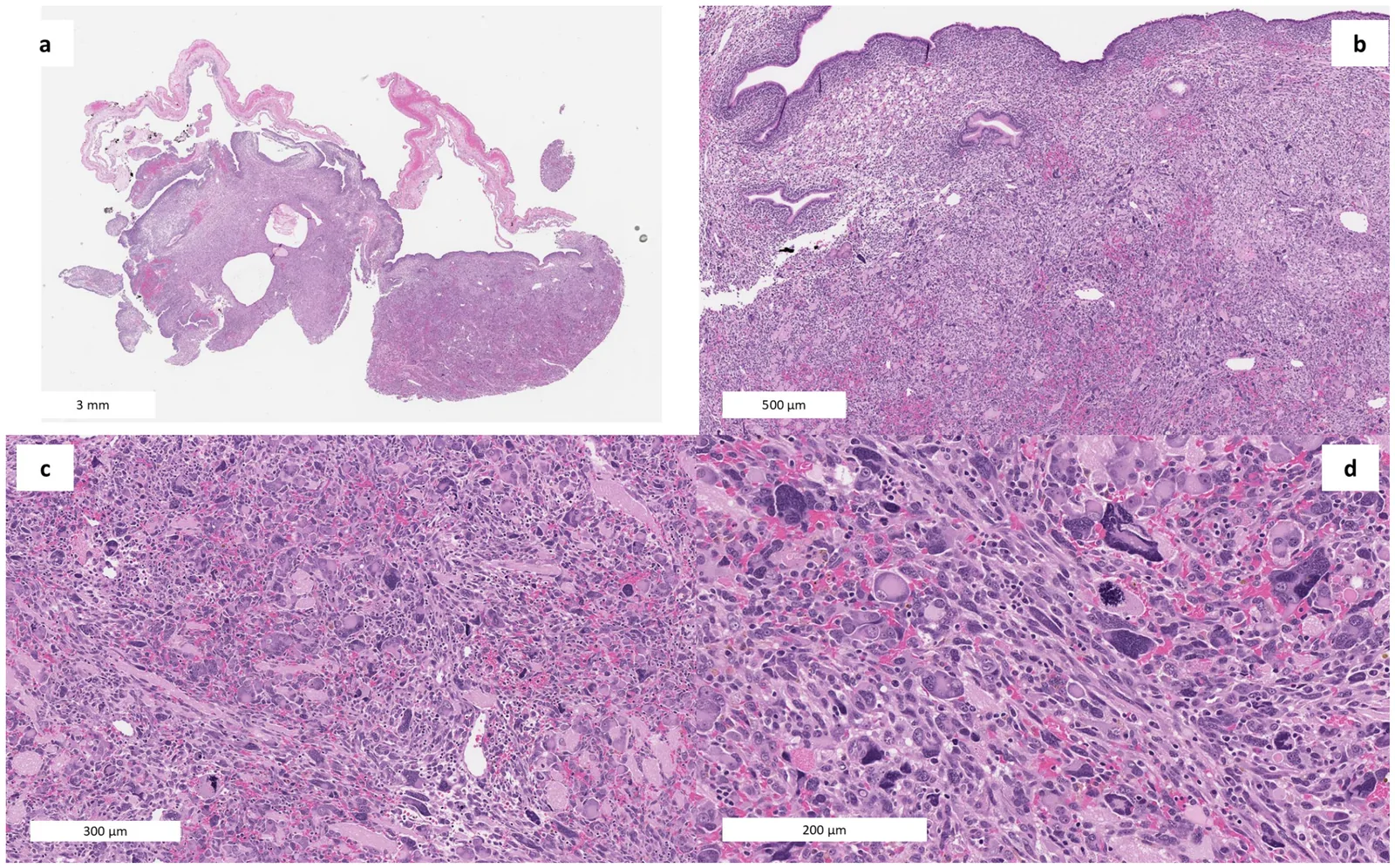
(**a**–**d**): Representative archival histopathological appearances of **mixed cystic and solid pleuropulmonary blastoma with anaplasia (PPB type II with anaplasia)** from cases of A.N. The tumor is a high-grade primitive multipatterned sarcoma with residual microscopic cystic foci of type I PPB (**a**,**b**; H&E, ×0.5, ×4). Residual epithelial-lined cystic foci with a subepithelial cambium layer of primitive small cells (type I component; **b**) merge with an adjacent high-grade solid sarcomatous mass composed of highly anaplastic cells with markedly enlarged pleomorphic nuclei and atypical mitotic figures (**c**,**d**; H&E, ×10, ×20). Archival cases provided by A.N.; these images are presented for illustrative purposes and do not represent additional cases included in the scoping-review dataset.

**Figure 9:**
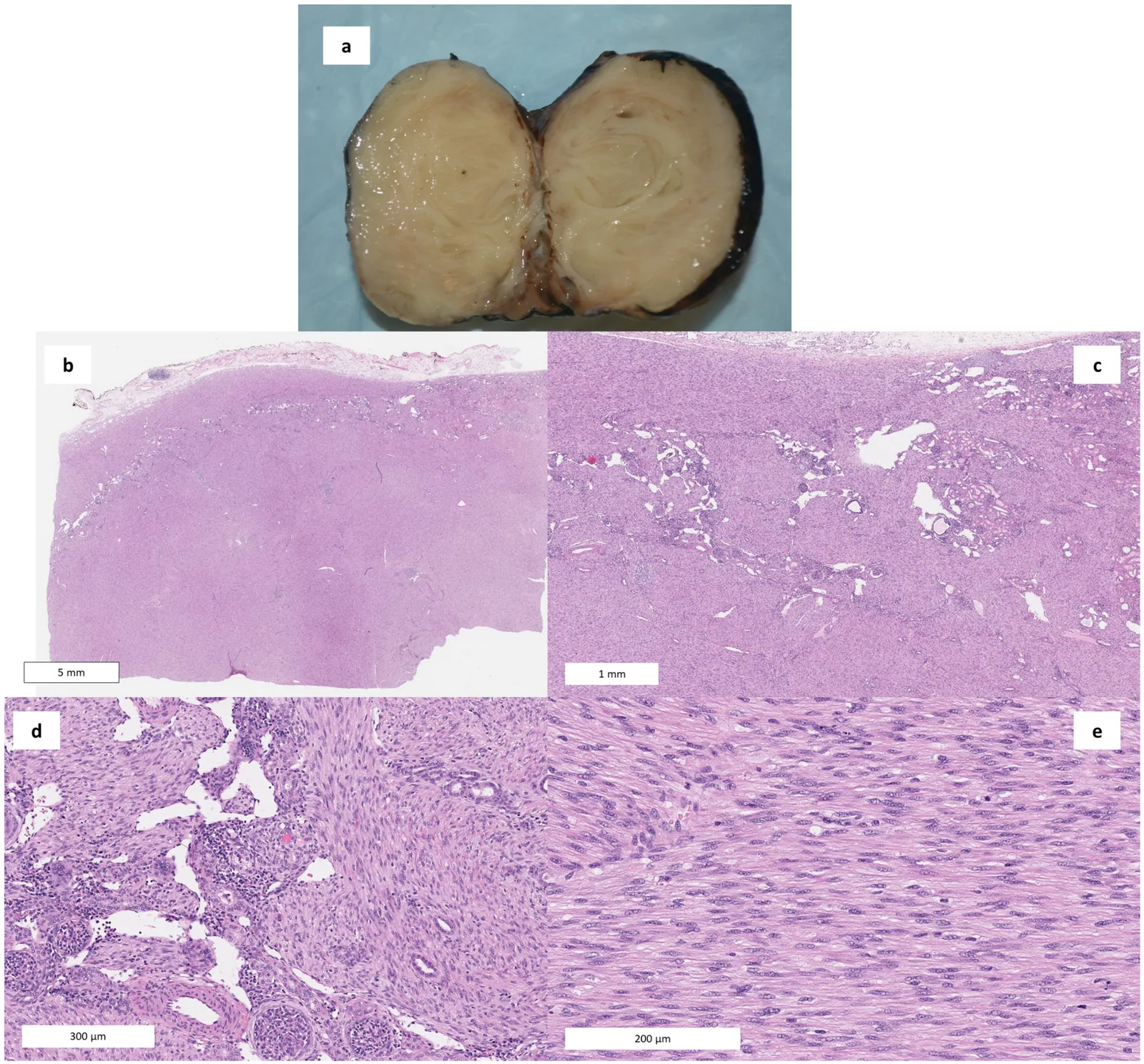
Representative archival gross and histopathological appearances of congenital mesoblastic nephroma from a case of A.N. Grossly, it appears as a solid, firm mass with a whorled, tan-gray cut surface (**a**). Congenital mesoblastic nephroma presents as a spindle-cell neoplasm with infiltrative borders and entrapment of native renal tubules/glomeruli (**b**–**d**; H&E, ×2, ×4, ×10). Areas with classic morphology demonstrate bland, low-cellularity fascicles with rare mitoses (**e**; H&E, ×20). This archival case is presented for illustrative purposes only and was not included as an additional case in the scoping-review dataset.

**Figure 10:**
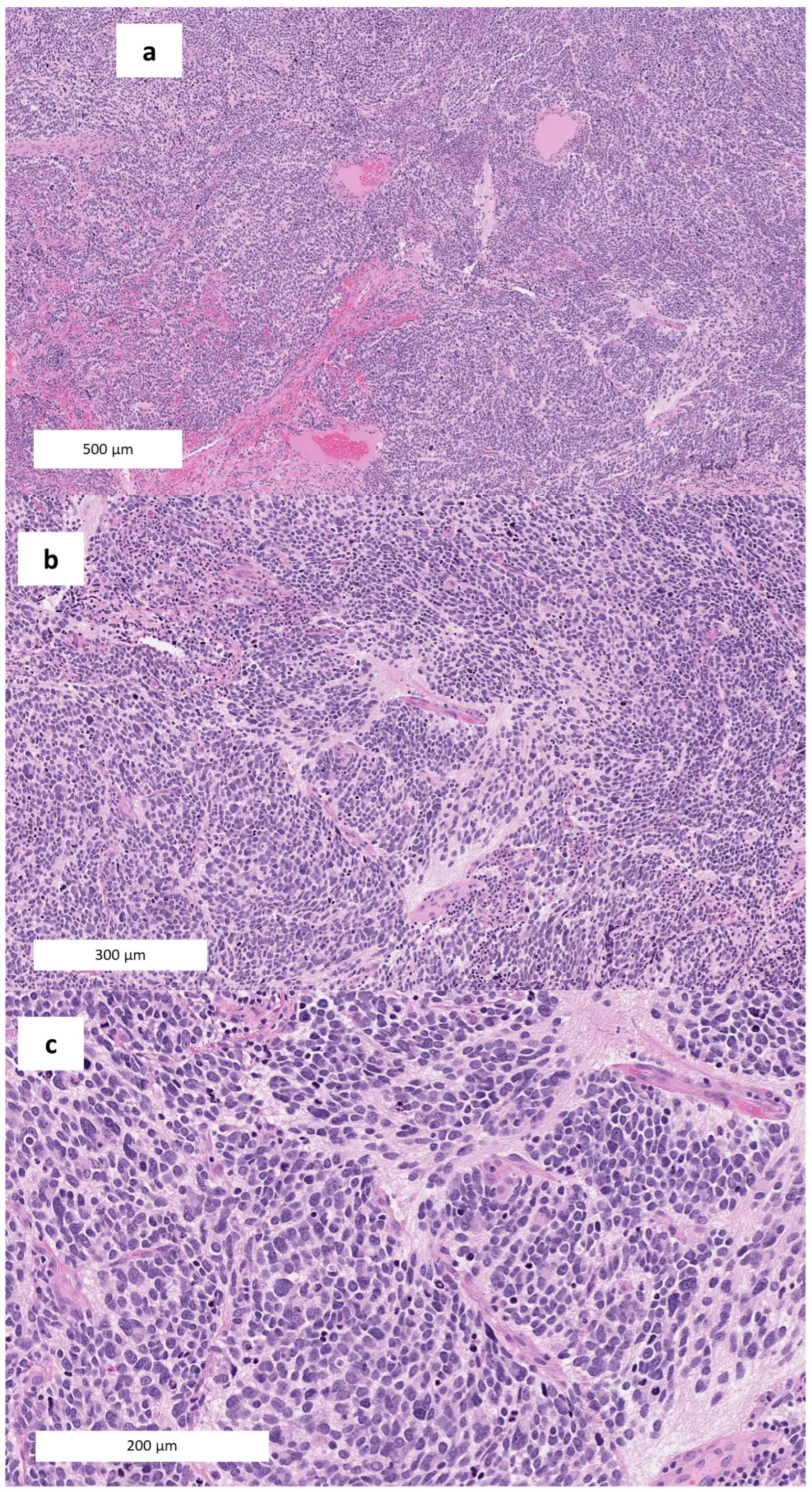
Representative archival histopathological appearances of **neuroblastoma** from cases of A.N. The tumor is a Schwannian stroma-poor neuroblastoma, composed of solid sheets, nests, and lobules of small round blue cells separated by delicate fibrovascular septa. The neoplastic cells are small round-to-oval neuroblasts with scant, poorly defined cytoplasm, a high nuclear-to-cytoplasmic ratio, hyperchromatic nuclei, and inconspicuous nucleoli. A characteristic fibrillary eosinophilic neuropil-like background is identified (**a**–**c**; H&E, ×4, ×10, and ×20, respectively). These archival cases are presented for illustrative purposes only and were not included as additional cases in the scoping-review dataset.

**Figure 11:**
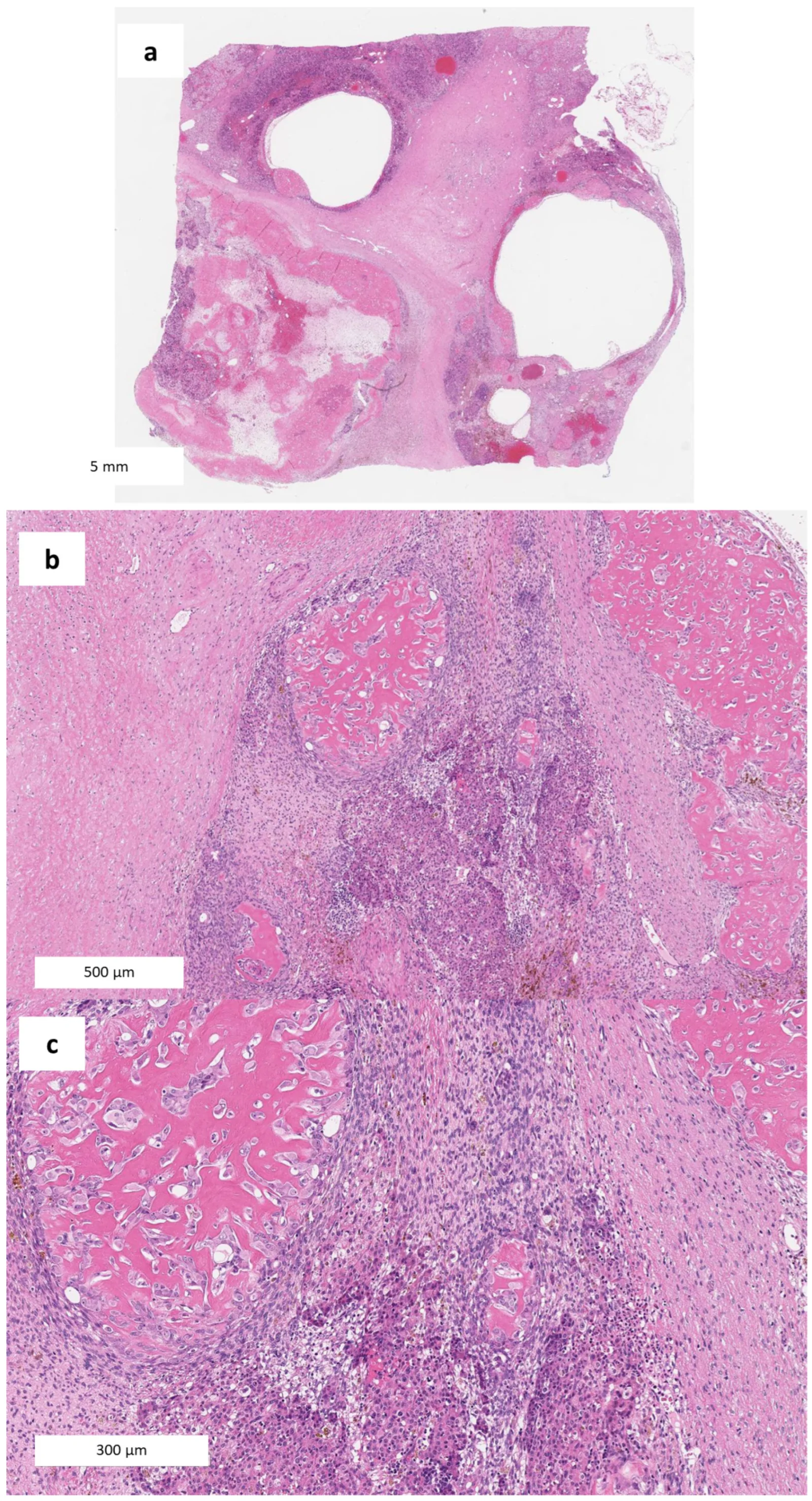
Representative archival histopathological appearances of **hepatoblastoma** from cases of A.N. The images (**a**–**c**) show a mixed epithelial–mesenchymal hepatoblastoma composed of sheets and trabeculae of primitive hepatoblastic cells, including fetal/embryonal-type areas, within a fibrous stroma. The tumor is associated with prominent heterologous osteoid and trabecular bone formation, with neoplastic cells surrounding and infiltrating the osseous matrix (H&E, ×0.5, ×4, and ×10, respectively). These archival cases are presented for illustrative purposes only and were not included as additional cases in the scoping-review dataset.

**Figure 12:**
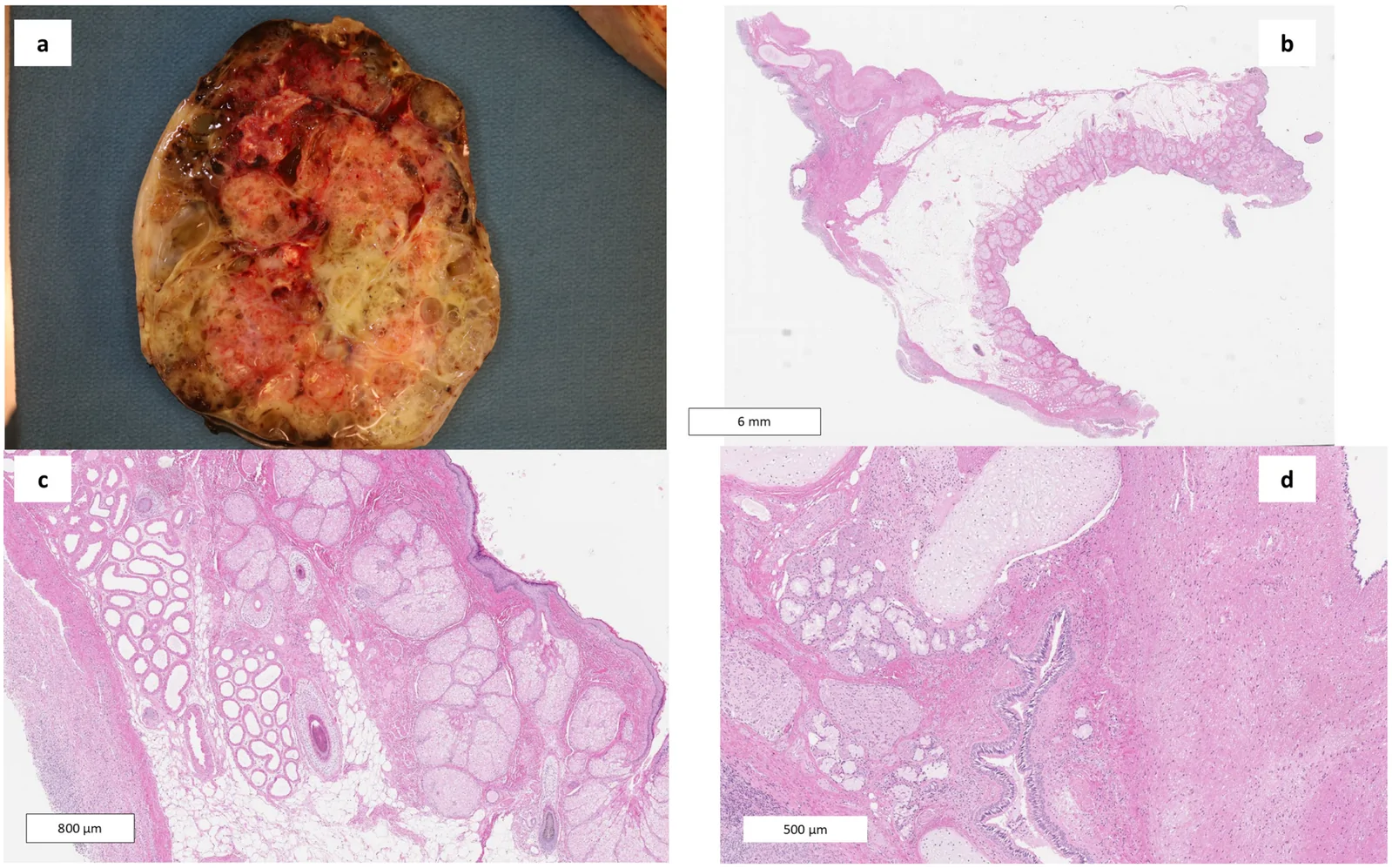
Representative archival appearances of **sacrococcygeal teratoma** from cases of A.N. (**a**): Grossly, the specimen consists of a well-circumscribed, lobulated mass with a heterogeneous cut surface. The lesion is predominantly multicystic, containing multiple variably sized cystic spaces filled with clear to mucoid/gelatinous material. Intermixed solid areas are tan-white to yellow, with focal red-brown hemorrhagic and congested regions. (**b**–**d**): Histological sections showing layers of different tissues: adipose tissue, mature skin with sebaceous glands, hair follicles (**b**,**c**, H&E, ×0.4, ×2), cartilage, small nerves, and respiratory mucosa (**d**; H&E, ×10). These archival cases are presented for illustrative purposes only and were not included as additional cases in the scoping-review dataset.

**Table 1:** Clinicopathological characteristics of individual cases of occult neoplasms identified in sudden unexpected death in infancy.

Case	Study	Age/Sex	Presentation and Circumstances	Neoplasm, Site, and Size	Key Post-Mortem Findings and Proposed Mechanism	Causal Role in Death
1	[9]	32 days, M	Persistent residual right-lung radiographic shadowing had been present since birth. Died suddenly 5 days after hospital discharge; exact location and circumstances were not reported.	Pulmonary blastoma (historical biphasic terminology); Right lung, lower lobe.	Clearly visible. Entire right lower lobe replaced; tumour extended to the visceral pleura without penetration; histological vascular invasion. Mechanism: Not explicitly stated; presumed acute respiratory compromise related to extensive replacement of the right lower lobe.	Causal
2	[10]	5 months, F	Three-day history of dyspnoea, sore throat and weakness, with worsening dyspnoea and hacking cough. Found unresponsive by a family member in supine position on her bed on the third day of illness.	Acute myeloid leukaemia with pulmonary leukostasis and extramedullary infiltration; systemic acute myeloid leukaemia with predominant bilateral pulmonary and hepatic infiltration; multifocal/disseminated.	Microscopic only. Bronchi, bronchioles, alveolar septa and lumina, pulmonary vascular lumina, and liver; severe bilateral pulmonary involvement. Mechanism: Acute respiratory failure due to diffuse pulmonary leukostasis and extramedullary myeloblastic infiltration.	Causal
3	[11]	11 months, M	No preceding cardiac or systemic symptoms were reported. While crawling and playing on the floor under his mother’s observation, the infant suddenly collapsed; he was dead on arrival at hospital.	Solitary cardiac rhabdomyoma (cardiac hamartoma); anterior interventricular septum and anterolateral wall of the left ventricle; largest dimension 3 cm.	Clearly visible. Tumour located near the main bundle of His, protruding into the left ventricular cavity and appreciably narrowing the left ventricular outflow tract. Mechanism: Probable fatal conduction disturbance with sudden asystole due to proximity to the His bundle; left ventricular outflow obstruction with acute cardiac failure was also considered.	Causal
4	[12]	Newborn, F	Acute deterioration at 1 h of age with severe metabolic acidosis; treated as suspected sepsis despite normal inflammatory markers. Acute unexpected deterioration shortly after birth; required ventilation and died several hours later.	Congenital adrenal neuroblastoma; left adrenal gland; largest dimension 3 cm.	Clearly visible. Left adrenal gland; tumour reported as confined to the adrenal gland. Mechanism: Possible catecholamine or other tumour-mediated metabolic/haemodynamic effect mimicking sepsis. Competing findings: Sepsis was clinically suspected, but inflammatory markers were normal; incomplete perinatal information and absence of blood-pressure and placental data limited interpretation.	Contributory
5	[12]	2.5 months, M	Swollen abdomen assessed by a general practitioner 13 days before death and considered a probable incidental umbilical hernia. Sudden collapse at home; confirmed dead on arrival at hospital.	Congenital mesoblastic nephroma; left kidney; largest dimension 10 cm.	Clearly visible. Large renal tumour with extensive intra-abdominal haemorrhage. Mechanism: Fatal intra-abdominal haemorrhage from a large necrotic renal tumour.	Causal
6	[12]	4 months, F	Poor feeding and irritability for 3 days; fever, dyspnoea, bruising and oro-nasal bleeding on presentation. Rapid deterioration after presentation to the emergency department; death occurred within minutes.	Acute leukaemia, not otherwise specified; systemic; lymph nodes, liver and spleen; multifocal/disseminated.	Clearly visible. Widespread lymphadenopathy and hepatosplenomegaly. Mechanism: Not specifically stated; likely acute marrow failure with haemorrhagic and/or infectious complications.	Contributory
7	[12]	4.5 months, M	No preceding symptoms. Suddenly stopped breathing during a feed.	Cardiac fibroma; heart; left ventricular outflow tract; largest dimension 5 cm.	Clearly visible. Large intracardiac tumour obstructing the left ventricular outflow tract. Mechanism: Acute cardiac failure and/or fatal arrhythmia related to severe left ventricular outflow obstruction.	Causal
8	[13]	Newborn, M	Failed to establish spontaneous respiration immediately after birth. Severe cardiorespiratory compromise from birth; death certified after 1 h of unsuccessful resuscitation.	Congenital mature intrapericardial teratoma; intrapericardial, attached by a pedicle to the intrapericardial ascending aorta; largest dimension 7 cm.	Clearly visible. Pericardial cavity, heart and both lungs; major pulmonary and cardiac compression. Mechanism: Cardiorespiratory failure caused by severe cardiac and pulmonary compression, associated pericardial effusion and pulmonary hypoplasia/collapse.	Causal
9	[13]	Newborn, M	Immediate severe respiratory distress due to a large anterior cervical mass. Upper airway obstruction began immediately after birth; intubation and tracheotomy were unsuccessful and death occurred after approximately 1 h.	Congenital cervical teratoma with mature and immature elements; anterior cervical region with marked tracheal compression and deviation; largest dimension 10 cm.	Clearly visible. Upper airway and lungs; severe tracheal compression/kinking and pulmonary hypoplasia. Mechanism: Fatal upper-airway obstruction caused by severe tracheal compression and kinking, exacerbated by pulmonary hypoplasia.	Causal
10	[13]	Newborn, F	Profuse bleeding from a large sacrococcygeal mass during delivery, followed by cardiorespiratory collapse. Bleeding began during vaginal delivery; cardiac arrest occurred after hospital transfer and death occurred during attempted surgical excision.	Congenital sacrococcygeal teratoma with mature and immature elements; sacrococcygeal region, type I; largest dimension 10 cm.	Clearly visible. External sacrococcygeal soft tissues and major blood vessels; traumatic rupture with massive haemorrhage. Mechanism: Acute traumatic haemorrhage from the tumour during vaginal delivery, resulting in hypovolaemic arrest and death.	Causal
11	[14]	3 months, F	No preceding symptoms. Suddenly collapsed in her mother’s arms at home after the evening feed; dead on arrival at hospital despite prolonged resuscitation. Routine paediatric examinations up to 2 months of age were normal.	Primary low-grade cardiac sarcoma, unclassifiable subtype; left ventricular free wall with diffuse myocardial and intracavitary extension; largest dimension 5 cm.	Clearly visible. Complete infiltration of the left ventricular myocardium and chamber, with mitral valve deformation and obstruction of left ventricular filling. Mechanism: Fatal ventricular fibrillation combined with tumour-related restrictive cardiomyopathy and obstruction of left ventricular filling.	Causal
12	[15]	24 days, M	No symptoms were reported after discharge; the infant appeared healthy until death. Died suddenly and unexpectedly in the crib.	Multifocal capillary hemangiomas of the mitral and tricuspid valves; mitral and tricuspid valves.	Clearly visible. Mitral and tricuspid valve leaflets; no involvement of the conduction system. Mechanism: Not directly demonstrated; a possible arrhythmic or haemodynamic effect of multifocal valvular haemangiomas was implied. Competing findings: Mild septal hypertrophy and patent foramen ovale were known, but no obstructive lesion or conduction-system abnormality was found.	Contributory
13	[15]	35 days, M	No preceding symptoms. Died suddenly and unexpectedly in the crib.	Solitary capillary hemangioma of the tricuspid valve; medial cusp of the tricuspid valve; largest dimension 1.5 mm.	Clearly visible. Tricuspid valve; no involvement of the cardiac conduction system. Mechanism: No specific fatal mechanism was demonstrated. Competing findings: Strong family history of unexplained infant death; the lesion was extremely small, with no obstruction or conduction-system involvement and no other convincing tumour-related lethal mechanism.	Incidental
14	[16]	1 month, M	Runny nose, mild wheezing and diarrhoea during the 2 days before death; increased sleepiness and fussiness, but feeding remained satisfactory. Found dead in a shared adult bed after a sleep period.	Multifocal infantile hepatic hemangioma (historically infantile hemangioendothelioma type 1); liver, multifocal; largest dimension 2 cm.	Clearly visible. Diffuse hepatic involvement with marked hepatomegaly, high-flow vascular shunting, diaphragmatic elevation and secondary cardiopulmonary compromise. Mechanism: High-output congestive cardiac failure due to arteriovenous and arterioportal shunting, with a secondary restrictive respiratory effect from the enlarged liver and diaphragmatic incursion.	Contributory
15	[17]	8 months, M	No preceding symptoms. Approximately 15 min after bottle feeding, the infant became cyanotic and lost consciousness. Sudden cyanosis and loss of consciousness at home shortly after bottle feeding; transported immediately to hospital and arrived without signs of life.	Cardiac fibroma; anterior and inferior free wall of the left ventricle; largest dimension 4.7 cm.	Clearly visible. Large intramural left-ventricular mass deforming the ventricular wall and compressing the ventricular cavity. Mechanism: Cardiogenic shock from severe ventricular-cavity compression; a fatal ventricular arrhythmia or conduction disturbance was also biologically plausible but not directly demonstrated.	Contributory
16	[18]	2.5 months, F	No acute preceding symptoms were reported. Found lifeless by her father in the parental bed, lying on a pillow beside her sleeping mother. Routine preventive medical examinations before death.	Multifocal microscopic cardiac rhabdomyoma; heart, multifocal microscopic intramyocardial foci; precise cardiac sites not specified.	Microscopic only. Possible involvement of the cardiac conduction system was proposed but not demonstrated. Mechanism: Acute dysrhythmia from presumed involvement of the cardiac conduction system. Competing findings: The infant was sleeping in an adult bed on a pillow beside her mother, creating a potentially hazardous sleep environment.	Contributory
17	[19]	2 days, F	No preceding symptoms were reported; the neonate was unexpectedly found completely cyanosed during the night on the second postnatal day. Resuscitation was initiated but was unsuccessful.	Cavernous hemangioma of the tricuspid valve; tricuspid valve; largest dimension 8 mm.	Clearly visible. Tricuspid valve; possible aggravation of right-sided haemodynamics through tricuspid regurgitation was proposed but not demonstrated. Mechanism: Fatal circulatory failure caused by severe hypoplasia of the left-sided cardiac structures; the tricuspid haemangioma may have worsened haemodynamics by producing tricuspid regurgitation. Competing findings: Severe hypoplastic left heart syndrome was a sufficient and more convincing primary cause of death.	Contributory
18	[20]	1 month, F	No preceding symptoms. The infant was suddenly found pale, unresponsive and not breathing while in a baby carrier. Shortly after the family entered a restaurant, the mother moved the baby carrier in the restroom and found the infant pale, unresponsive and apnoeic.	Cardiac fibroma; posterior wall of the left ventricle, extending to the ventricular septum and apex; largest dimension 4 cm.	Clearly visible. The tumour compressed and narrowed both ventricular outflow tracts and compressed the myocardium of the left bundle branch. Mechanism: Combined impairment of ventricular blood flow and fatal cardiac arrhythmia caused by overstretching and compression of the left bundle branch.	Causal
19	[21]	5 months, F	No preceding respiratory, neurological or systemic symptoms were reported. Placed to sleep at approximately 22:00 and found unresponsive at 06:45 in a recently purchased playpen adapted as a crib. Routine paediatric follow-up had not identified tuberous sclerosis.	Tuberous sclerosis complex with multifocal cardiac rhabdomyomas and subependymal giant cell astrocytoma; heart and central nervous system; largest dimension 1.5 cm.	Clearly visible. Cardiac lesions involved intramural, subendocardial and subpericardial myocardium and could potentially affect the conduction system. Mechanism: Possible fatal arrhythmia from cardiac rhabdomyomas or fatal seizure related to extensive cortical tubers/subependymal tumour, with a possible concurrent positional or sleep-environment asphyxial mechanism. Competing findings: Prone sleeping and a soft sloping mattress represented relevant environmental factors.	Contributory
20	[22]	3 months, M	Persistent cough associated with a recent upper respiratory tract infection; no other acute symptoms were reported. Fed at 08:30 and placed in his crib; found lifeless at 12:15.	Solitary microscopic cardiac neuroma/neurofibroma-like hamartomatous lesion; region of the atrioventricular node; largest dimension 0.75 mm.	Microscopic only. Located adjacent to the atrioventricular node; the atrioventricular node and bundle of His were structurally normal. Mechanism: Unexplained sudden infant death. No arrhythmic or obstructive mechanism attributable to the neural lesion was demonstrated. Competing findings: Recent upper respiratory tract infection with persistent cough and positive pleural-fluid culture without histological pneumonia.	Incidental
21	[23]	2 months, M	A grade 5 holosystolic murmur radiating to the axilla and back was detected during a routine well-baby examination one day before death. The infant suddenly collapsed in his mother’s arms the following day.	Multifocal cardiac myxoma; multifocal cardiac involvement of both atria, aortic, mitral and tricuspid valves, left ventricular endocardium and membranous septum; largest dimension 2.3 cm.	Clearly visible. Near-complete aortic-valve obstruction with a residual orifice of 0.1 cm; marked mitral-valve obstruction; diffuse tricuspid-valve involvement. Mechanism: Mechanical and haemodynamic disturbance due to severe multivalvular obstruction; a conduction-related effect was also considered possible. Embolic death was excluded.	Causal
22	[24]	3.5 months, F	None reported after discharge. Found dead by her father in a prone position in the parents’ bed at approximately 3.5 months of age.	Multifocal infantile hepatic vascular tumour, historically termed type II infantile hepatic hemangioendothelioma; liver, multifocal lesions involving both lobes; largest dimension 2 cm.	Clearly visible. Liver parenchyma; no direct compression of a vital structure and no evidence of cardiac failure. Mechanism: Unexplained sudden infant death; a possible degree of tumour-related cardiovascular compromise was discussed but not demonstrated. Competing findings: Prone sleeping in the parents’ bed, minor repeated milk aspiration changes, and a previously resolved systolic murmur.	Contributory
23	[25]	4 months, F	Two episodes of vomiting two days before death; otherwise no illness or complaint was known. Sudden and unexpected death; the precise location and circumstances of discovery were not reported.	Microscopic septal cardiac fibroma involving the atrioventricular conduction region; cardiac fibrous body/interventricular septal region adjacent to and compressing the atrioventricular node.	Microscopic only. Atrioventricular node and adjacent conduction tissue. Mechanism: Possible fatal conduction disturbance or arrhythmia caused by compression of the atrioventricular node, potentially acting together with an accessory Mahaim fibre and other conduction-system abnormalities.	Contributory
24	[26]	5 months, F	None reported. Found unresponsive during a nap in an adult bed at her caregiver’s home; she had been last known alive two hours earlier.	Multifocal microscopic left ventricular rhabdomyoma; multifocal microscopic involvement of the left ventricular wall, trabeculae and immediately subendocardial myocardium.	Microscopic only. Left ventricular subendocardial myocardium; a potential arrhythmic substrate was inferred, but direct conduction-system involvement was not documented. Mechanism: Presumed fatal cardiac arrhythmia caused by microscopic rhabdomyoma.	Contributory
25	[27]	7 weeks, F	A skin nodule and abdominal distension developed after the 4-week check-up. On the day of death she suddenly developed gasping respiration while feeding, followed by respiratory and cardiac arrest at home.	KMT2A-MLLT1-rearranged infantile B-cell acute lymphoblastic leukaemia; systemic disease involving bone marrow, skin, liver, spleen, kidneys, pancreas, gastrointestinal tract, uterus, lymph nodes and vascular lumina of multiple organs including heart and lungs; multifocal/disseminated.	Clearly visible. Bone marrow and widespread visceral and intravascular infiltration, particularly pulmonary vascular involvement. Mechanism: Severe anaemia, hypoxaemia from widespread intravascular pulmonary blast infiltration and hyperkalaemia related to tumour lysis syndrome.	Causal
26	[28]	6 months, F	None reported before the sudden collapse. Became unresponsive after crying; emergency services documented ventricular fibrillation.	Large septal cardiac fibroma; interventricular septum and left ventricle, extending from apex to base and affecting both ventricular outflow tracts and the atrioventricular conduction system; largest dimension 4.5 cm.	Clearly visible. Left ventricular cavity, right ventricular infundibulum, mitral valve, aortic outflow, atrioventricular node, His bundle and both bundle branches. Mechanism: Left ventricular outflow obstruction and severe conduction-system compression causing bradyarrhythmia/ventricular fibrillation and acute haemodynamic failure.	Causal
27	[29]	2 months, F	None reported. Became unresponsive and was taken to an emergency department; the precise location and activity at collapse were not reported.	Multifocal cardiac Purkinje-cell tumour/histiocytoid cardiomyopathy; multifocal lesions in left and right endocardium, interatrial septum, both atrial walls and near the sinoatrial and atrioventricular nodes.	Microscopic only. Cardiac conduction system, including lesions close to the sinoatrial and atrioventricular nodes. Mechanism: Fatal cardiac electrical instability caused by diffuse involvement and compression of the specialised conduction system.	Contributory
28	[30]	6 months, F	A slight cough occurred after feeding approximately one hour before she was found dead; otherwise, no concern had been reported. Found dead in her cot by her father approximately one hour after feeding.	Mixed fetal and embryonal epithelial hepatoblastoma; right hepatic lobe, occupying most of the lobe; largest dimension 12 cm.	Clearly visible. Liver and peritoneal cavity; extensive tumour necrosis and haemorrhage with 200 mL haemoperitoneum, although no rupture site was identified. Mechanism: Likely fatal infection in a markedly underweight infant with advanced malignancy; haemoperitoneum and tumour necrosis were additional possible contributors, but no bleeding source was demonstrated. Competing findings: Focal bilateral bronchopneumonia and chronic tracheal inflammation.	Contributory
29	[30]	3.5 months, M	None reported. Found dead in a prone position in his pram approximately 30 min after breastfeeding and being put to sleep.	Thoracic extradural spinal lipoma with recent haemorrhage; thoracic extradural spinal canal, extending for approximately 7 cm below the cervical region; largest dimension 7 cm.	Clearly visible. Thoracic spinal canal adjacent to the spinal cord; no direct involvement of the phrenic nerve or demonstrable medullary compression. Mechanism: Unexplained sudden infant death. The authors considered but could not demonstrate increased intraspinal pressure acting with prone positioning to inhibit respiration. Competing findings: Prone sleeping position and recent haemorrhage within the lipoma.	Incidental
30	[31]	1 month, F	Lethargy and poor feeding. Found unresponsive the morning after medical review; resuscitation attempts were unsuccessful.	Precursor B-cell acute lymphoblastic leukaemia; systemic leukaemia with distension of visceral and cerebral vessels by leukaemic cells; multifocal/disseminated.	Microscopic only. Visceral and cerebral microvasculature. Mechanism: Fatal microvascular obstruction from the extreme intravascular leukaemic burden.	Causal
31	[31]	2 months, M	Tachycardia, tachypnoea and fever of 39.9 °C on the day of death. Cardiorespiratory arrest later the same day; resuscitation was unsuccessful.	Acute myeloid leukaemia with diffuse multiorgan and myocardial infiltration; systemic acute myelogenous leukaemia with hepatosplenomegaly, lymphadenopathy and extensive multiorgan infiltration including myocardium; multifocal/disseminated.	Clearly visible. Myocardium and systemic organs; cardiac infiltration caused tissue disruption, oedema, necrosis and haemorrhage. Mechanism: Diffuse myocardial infiltration and injury causing acute cardiac failure.	Causal
32	[32]	16 weeks, M	No overt illness in the week before death. Home video taken within 24 h of death showed a prominent abdomen and subtle lower-chest intercostal recession. Found moribund in his crib by his father.	Precursor B-cell acute lymphoblastic leukaemia with diffuse multiorgan and myocardial infiltration; systemic disease involving bone marrow, heart, lungs, liver, kidneys, stomach, intestines, appendix, pancreas, thyroid, salivary gland, lymph nodes, spleen, adipose tissue and multiple vascular beds; multifocal/disseminated.	Clearly visible. Myocardium, coronary and systemic vessels, pulmonary interstitium and multiple abdominal organs. Mechanism: Probable fatal arrhythmia caused by diffuse myocardial infiltration, potentially compounded by pulmonary infiltration, restricted respiratory expansion from organomegaly, leukocytosis and hyperviscosity within coronary vessels.	Causal

Abbreviations: F, female; M, male.

**Table 2:** Summary characteristics of the included infant cases (*n* = 32).

Domain	Variable	Cases, *n*/*N* (%) or Summary
**Demographic characteristics**	Male	15/32 (46.9%)
	Female	17/32 (53.1%)
	Age, median (range)	2.5 months (birth–11 months)
	Neonatal (<28 days)	6/32 (18.8%)
	28 days to <3 months	11/32 (34.4%)
	3 to <6 months	11/32 (34.4%)
	6 to <12 months	4/32 (12.5%)
**Clinical presentation and circumstances**	No preceding symptoms or signs reported	15/32 (46.9%)
	Any preceding symptoms or signs reported	17/32 (53.1%)
	Recent healthcare contact reported	21/32 (65.6%)
	Found unresponsive, moribund, or dead	15/32 (46.9%)
	Witnessed collapse or acute deterioration	15/32 (46.9%)
	Collapse during or within 1 h after feeding	6/32 (18.8%)
	Resuscitation attempted	20/32 (62.5%)
**Neoplasm category**	Cardiovascular/thoracic	16/32 (50.0%)
	Haematological/lymphoid	6/32 (18.8%)
	Germ-cell/embryonal	3/32 (9.4%)
	Hepatic/gastrointestinal	3/32 (9.4%)
	Adrenal/endocrine	1/32 (3.1%)
	Renal/genitourinary	1/32 (3.1%)
	Nervous system	1/32 (3.1%)
	Disseminated/multisystem	1/32 (3.1%)
**Pathological characteristics**	Benign	16/32 (50.0%)
	Intermediate/uncertain or tumour-like	6/32 (18.8%)
	Malignant	10/32 (31.3%)
	Multifocal or disseminated disease	14/32 (43.8%)
	Clearly visible at gross examination	25/32 (78.1%)
	Microscopic only	7/32 (21.9%)
	Largest tumour dimension reported	20/32 (62.5%)
	Largest dimension, median (range)	42.5 mm (0.75–120 mm), *n* = 20
**Proposed mechanism of death**	Arrhythmia or conduction-system disturbance	14/32 (43.8%)
	Mechanical obstruction or compression	9/32 (28.1%)
	Cardiac failure or haemodynamic compromise	14/32 (43.8%)
	Haemorrhage, rupture, or exsanguination	4/32 (12.5%)
	Respiratory failure or pulmonary involvement	9/32 (28.1%)
	Systemic haematological effects or diffuse infiltration	6/32 (18.8%)
	Neurological or seizure-related mechanism	2/32 (6.3%)
	Infectious complication	2/32 (6.3%)
	Mechanism uncertain or not demonstrated	5/32 (15.6%)
**Review causal assessment**	Causal	16/32 (50.0%)
	Contributory	13/32 (40.6%)
	Incidental	3/32 (9.4%)
**Ancillary diagnostic methods**	Immunohistochemistry reported	11/32 (34.4%)
	Molecular or genetic testing reported	3/32 (9.4%)
	Post-mortem imaging reported	2/32 (6.3%)

Data are presented as *n*/*N* (%) unless otherwise indicated; the denominator was 32 cases for all categorical variables. Clinical presentation, circumstances of death, and proposed mechanisms were coded by the review team from the original case descriptions. Categories relating to clinical circumstances, proposed mechanisms, and ancillary diagnostic methods were not mutually exclusive; therefore, percentages may exceed 100%. Molecular or genetic testing included tumour-directed molecular/cytogenetic analyses and testing for inherited conditions.

## Data Availability

The data supporting the findings of this study are available within the article and its Supplementary Materials. No new primary data were generated during this study.

## Supplementary Materials

The following supporting information can be downloaded at https://www.mdpi.com/article/10.3390/diagnostics16193189/s1. File S1: Complete electronic search strategies for PubMed/MEDLINE and Scopus; Table S1: Individual case characteristics of occult neoplasms in sudden unexpected death in infancy, including the data dictionary and controlled lists used for data extraction. File S2: PRISMA-ScR_Checklist_FINAL. Ref. [35] is cited in the supplementary materials.

## Abbreviations

The following abbreviations are used in this manuscript:

PRISMA-ScR	Preferred Reporting Items for Systematic Reviews and Meta-Analyses Extension for Scoping Reviews
SUDI	Sudden unexpected death in infancy
SIDS	Sudden infant death syndrome
NGS	Next-generation sequencing
ECG	Electrocardiogram
WHO	World Health Organization
