# Supplementary material for: Occult Neoplasms in Sudden Unexpected Death in Infancy: A Scoping Review of Post-Mortem Findings
Source: Diagnostics (Basel). 2026 Sep 30;16(19):3189. doi: 10.3390/diagnostics16193189 (PMC13664608; doi:10.3390/diagnostics16193189)
Supplement: Supplementary file 1 [file diagnostics-16-03189-s001.zip › File S2_PRISMA-ScR_Checklist_FINAL.pdf]

PRISMA-ScR Checklist (Completed)

Manuscript: Occult Neoplasms in Sudden Unexpected Death in Infancy: A Scoping Review of Post-Mortem Findings

Checklist prepared for journal submission. Items 12 and 16 are optional in PRISMA-ScR; both are reported in this review.

Section	Topic	Item	PRISMA-ScR reporting requirement (paraphrased)	Reported on page(s)
TITLE	Title	1	Identify the report explicitly as a scoping review.	p. 1
ABSTRACT	Structured summary	2	Provide a structured summary covering the review context, objective, eligibility, evidence sources, charting approach, main results, and conclusions.	pp. 1–2
INTRODUCTION	Rationale	3	Explain the review rationale in light of existing knowledge and why a scoping-review approach is appropriate.	pp. 2–3
INTRODUCTION	Objectives	4	State the review questions/objectives explicitly and define the key elements used to frame the scope.	p. 3
METHODS	Protocol and registration	5	State whether a protocol exists, whether it is accessible, and whether the review was registered.	p. 3
METHODS	Eligibility criteria	6	Specify source and report characteristics used for inclusion/exclusion and provide the rationale for eligibility decisions.	pp. 3–4
METHODS	Information sources	7	Describe all information sources used and report the date of the most recent search.	p. 4; Supplementary File S1
METHODS	Search	8	Provide a reproducible electronic search strategy, including database-specific syntax and limits where applicable.	Supplementary File S1, pp. 1–20
METHODS	Selection of sources of evidence	9	Describe the screening and eligibility process used to select sources for inclusion.	p. 4
METHODS	Data charting process	10	Describe how data were charted/extracted, including reviewer independence or duplication and use of a standardised form.	p. 4
METHODS	Data items	11	List the variables sought from included sources and indicate how they were defined or standardised.	p. 4; Supplementary Table S1
METHODS	Critical appraisal of individual sources (optional)	12	If appraisal was performed, describe the appraisal tools, process, and how appraisal informed interpretation.	p. 4
METHODS	Synthesis of results	13	Describe how charted data were summarised, grouped, and synthesised.	pp. 4–5
RESULTS	Selection of sources of evidence	14	Report screening and inclusion numbers, reasons for full-text exclusion, and present the selection flow.	p. 5; Figure 1
RESULTS	Characteristics of sources of evidence	15	Present the relevant characteristics of the included evidence and provide citations.	pp. 5–16; Table 1
RESULTS	Critical appraisal within sources (optional)	16	Present the results of the critical appraisal when appraisal was undertaken.	p. 21
RESULTS	Results of individual sources of evidence	17	Present relevant case- or source-level findings for the included evidence.	pp. 7–16; Table 1; Supplementary Table S1
RESULTS	Synthesis of results	18	Present the mapped and synthesised findings in relation to the review questions and objectives.	pp. 17–21; Table 2
DISCUSSION	Summary of evidence	19	Summarise the principal findings, relate them to the review objectives, and discuss their relevance and implications.	pp. 21–24
DISCUSSION	Limitations	20	Discuss limitations of the scoping-review process and of the available evidence.	p. 24
DISCUSSION	Conclusions	21	Provide an overall interpretation of the findings and implications for practice, policy, or future research.	p. 24
FUNDING	Funding	22	Describe funding or support for the included evidence and the scoping review, including the role of funders when applicable.	p. 25

Reference: Tricco AC, Lillie E, Zarin W, et al. PRISMA Extension for Scoping Reviews (PRISMA-ScR): Checklist and Explanation. Ann Intern Med. 2018;169(7):467–473. doi:10.7326/M18-0850.

Note: The reporting-requirement text above is paraphrased; item numbering and section/topic labels follow PRISMA-ScR.
