# Supplementary material for: Occult Neoplasms in Sudden Unexpected Death in Infancy: A Scoping Review of Post-Mortem Findings
Source: Diagnostics (Basel). 2026 Sep 30;16(19):3189. doi: 10.3390/diagnostics16193189 (PMC13664608; doi:10.3390/diagnostics16193189)
Supplement: Supplementary file 1 [file diagnostics-16-03189-s001.zip › Supplementary_File_S1.pdf]

Supplementary File S1

Complete electronic search strategies for PubMed/MEDLINE and Scopus

Occult neoplasms in sudden unexpected death in infancy

PubMed/MEDLINE search strategies

The following strategies are formatted for direct use in the PubMed search box. MeSH terms are combined with Title/Abstract terms, and automatic MeSH explosion is retained.

PUBMED-01 | Comprehensive autopsy-focused neoplasm search

**Database**	PubMed/MEDLINE
**Purpose**	Primary, site-independent search intended to retrieve any benign, malignant, embryonal, haematological, or tumour-like neoplasm detected during a post-mortem investigation of sudden unexpected death. The MeSH term Neoplasms was searched without the noexp modifier and therefore automatically included narrower neoplasm concepts.
**Date searched**	July 19, 2026
**Records retrieved**	211 results

Exact search string

```
(
  "Sudden Infant Death"[Mesh]
  OR "Death, Sudden"[Mesh]
  OR "sudden unexpected death in infancy"[Title/Abstract]
  OR "sudden unexpected infant death"[Title/Abstract]
  OR "sudden unexplained death in infancy"[Title/Abstract]
  OR "sudden unexplained infant death"[Title/Abstract]
  OR "sudden infant death"[Title/Abstract]
  OR "unexpected infant death"[Title/Abstract]
  OR "sudden neonatal death"[Title/Abstract]
  OR "unexpected neonatal death"[Title/Abstract]
  OR "unexpected death"[Title/Abstract]
  OR "unexplained death"[Title/Abstract]
  OR "found dead"[Title/Abstract]
  OR "fatal collapse"[Title/Abstract]
  OR "cot death"[Title/Abstract]
  OR "sudden death"[Title/Abstract]
  OR SUDI[Title/Abstract]
  OR SUID[Title/Abstract]
  OR SUIDS[Title/Abstract]
  OR SIDS[Title/Abstract]
  OR SUDNIC[Title/Abstract]
)
AND
(
  "Infant"[Mesh]
  OR "Infant, Newborn"[Mesh]
  OR infant*[Title/Abstract]
  OR newborn*[Title/Abstract]
  OR neonat*[Title/Abstract]
  OR baby[Title/Abstract]
  OR babies[Title/Abstract]
  OR child*[Title/Abstract]
  OR pediatric*[Title/Abstract]
  OR paediatric*[Title/Abstract]
)
AND
(
  "Neoplasms"[Mesh]
  OR "Hamartoma"[Mesh]
  OR neoplasm*[Title/Abstract]
  OR neoplasia*[Title/Abstract]
  OR neoplastic[Title/Abstract]
  OR tumor*[Title/Abstract]
  OR tumour*[Title/Abstract]
  OR cancer*[Title/Abstract]
  OR oncolog*[Title/Abstract]
  OR malignan*[Title/Abstract]
  OR carcinoma*[Title/Abstract]
  OR adenocarcinoma*[Title/Abstract]
  OR sarcoma*[Title/Abstract]
  OR blastoma*[Title/Abstract]
  OR leukem*[Title/Abstract]
  OR leukaem*[Title/Abstract]
  OR lymphoma*[Title/Abstract]
  OR myeloma*[Title/Abstract]
  OR histiocyt*[Title/Abstract]
  OR germinoma*[Title/Abstract]
  OR teratoma*[Title/Abstract]
  OR hamartoma*[Title/Abstract]
  OR myofibroma*[Title/Abstract]
  OR papilloma*[Title/Abstract]
  OR glioma*[Title/Abstract]
  OR melanoma*[Title/Abstract]
)
AND
(
  "Autopsy"[Mesh]
```

```
  OR autops*[Title/Abstract]
  OR postmortem[Title/Abstract]
  OR "post-mortem"[Title/Abstract]
  OR "post mortem"[Title/Abstract]
  OR "postmortem examination"[Title/Abstract]
  OR "post-mortem examination"[Title/Abstract]
  OR "post mortem examination"[Title/Abstract]
  OR necrops*[Title/Abstract]
  OR forensic[Title/Abstract]
  OR medicolegal[Title/Abstract]
  OR "medico-legal"[Title/Abstract]
  OR "death investigation"[Title/Abstract]
  OR virtopsy[Title/Abstract]
)
```

PUBMED-02 | Cardiovascular, mediastinal, pulmonary, pleural, and other thoracic neoplasms

**Database**	PubMed/MEDLINE
**Purpose**	Supplementary search for cardiac, pericardial, vascular, mediastinal, thymic, pulmonary, pleural, bronchial, tracheal, and other thoracic neoplasms, including rare benign and malignant histological entities.
**Date searched**	July 19, 2026
**Records retrieved**	60 results

Exact search string

```
(
  "Sudden Infant Death"[Mesh]
  OR "Death, Sudden"[Mesh]
  OR "sudden unexpected death in infancy"[Title/Abstract]
  OR "sudden unexpected infant death"[Title/Abstract]
  OR "sudden unexplained death in infancy"[Title/Abstract]
  OR "sudden unexplained infant death"[Title/Abstract]
  OR "sudden infant death"[Title/Abstract]
  OR "unexpected infant death"[Title/Abstract]
  OR "sudden neonatal death"[Title/Abstract]
  OR "unexpected neonatal death"[Title/Abstract]
  OR "unexpected death"[Title/Abstract]
  OR "unexplained death"[Title/Abstract]
  OR "found dead"[Title/Abstract]
  OR "fatal collapse"[Title/Abstract]
  OR "cot death"[Title/Abstract]
  OR "sudden death"[Title/Abstract]
  OR SUDI[Title/Abstract]
  OR SUID[Title/Abstract]
  OR SUIDS[Title/Abstract]
  OR SIDS[Title/Abstract]
  OR SUDNIC[Title/Abstract]
)
AND
(
  "Infant"[Mesh]
  OR "Infant, Newborn"[Mesh]
  OR infant*[Title/Abstract]
  OR newborn*[Title/Abstract]
  OR neonat*[Title/Abstract]
  OR baby[Title/Abstract]
  OR babies[Title/Abstract]
  OR child*[Title/Abstract]
  OR pediatric*[Title/Abstract]
  OR paediatric*[Title/Abstract]
)
AND
(
  "Thoracic Neoplasms"[Mesh]
  OR "Heart Neoplasms"[Mesh]
  OR "Mediastinal Neoplasms"[Mesh]
  OR "Respiratory Tract Neoplasms"[Mesh]
  OR "Lung Neoplasms"[Mesh]
  OR "Pleural Neoplasms"[Mesh]
  OR "Vascular Neoplasms"[Mesh]
  OR "cardiac tumor"[Title/Abstract]
  OR "cardiac tumour"[Title/Abstract]
  OR "cardiac neoplasm"[Title/Abstract]
  OR "heart tumor"[Title/Abstract]
  OR "heart tumour"[Title/Abstract]
  OR "intracardiac tumor"[Title/Abstract]
  OR "intracardiac tumour"[Title/Abstract]
  OR "pericardial tumor"[Title/Abstract]
  OR "pericardial tumour"[Title/Abstract]
  OR "mediastinal mass"[Title/Abstract]
  OR "mediastinal tumor"[Title/Abstract]
  OR "mediastinal tumour"[Title/Abstract]
  OR "thymic tumor"[Title/Abstract]
  OR "thymic tumour"[Title/Abstract]
  OR thymoma*[Title/Abstract]
  OR "thoracic tumor"[Title/Abstract]
  OR "thoracic tumour"[Title/Abstract]
  OR "pulmonary tumor"[Title/Abstract]
  OR "pulmonary tumour"[Title/Abstract]
  OR "lung tumor"[Title/Abstract]
  OR "lung tumour"[Title/Abstract]
  OR "pleural tumor"[Title/Abstract]
  OR "pleural tumour"[Title/Abstract]
  OR "bronchial tumor"[Title/Abstract]
  OR "bronchial tumour"[Title/Abstract]
  OR "tracheal tumor"[Title/Abstract]
  OR "tracheal tumour"[Title/Abstract]
  OR "pleuropulmonary blastoma"[Title/Abstract]
  OR mesothelioma*[Title/Abstract]
  OR rhabdomyoma*[Title/Abstract]
  OR "cardiac fibroma"[Title/Abstract]
  OR "cardiac myxoma"[Title/Abstract]
  OR fibroelastoma*[Title/Abstract]
```

```
  OR angiomyoma*[Title/Abstract]
  OR hemangioma*[Title/Abstract]
  OR haemangioma*[Title/Abstract]
  OR lymphangioma*[Title/Abstract]
  OR "inflammatory myofibroblastic tumor"[Title/Abstract]
  OR "inflammatory myofibroblastic tumour"[Title/Abstract]
  OR "myofibroblastic sarcoma"[Title/Abstract]
  OR "cardiac sarcoma"[Title/Abstract]
  OR "pericardial teratoma"[Title/Abstract]
)
AND
(
  "Autopsy"[Mesh]
  OR autops*[Title/Abstract]
  OR postmortem[Title/Abstract]
  OR "post-mortem"[Title/Abstract]
  OR "post mortem"[Title/Abstract]
  OR "postmortem examination"[Title/Abstract]
  OR "post-mortem examination"[Title/Abstract]
  OR "post mortem examination"[Title/Abstract]
  OR necrops*[Title/Abstract]
  OR forensic[Title/Abstract]
  OR medicolegal[Title/Abstract]
  OR "medico-legal"[Title/Abstract]
  OR "death investigation"[Title/Abstract]
  OR virtopsy[Title/Abstract]
)
```

PUBMED-03 | Nervous system, head and neck, ocular, and neuroectodermal neoplasms

**Database**	PubMed/MEDLINE
**Purpose**	Supplementary search for central and peripheral nervous system, intracranial, spinal, head and neck, ocular, orbital, pineal, choroid plexus, and neuroectodermal neoplasms.
**Date searched**	July 19, 2026
**Records retrieved**	23 results

Exact search string

```
(
  "Sudden Infant Death"[Mesh]
  OR "Death, Sudden"[Mesh]
  OR "sudden unexpected death in infancy"[Title/Abstract]
  OR "sudden unexpected infant death"[Title/Abstract]
  OR "sudden unexplained death in infancy"[Title/Abstract]
  OR "sudden unexplained infant death"[Title/Abstract]
  OR "sudden infant death"[Title/Abstract]
  OR "unexpected infant death"[Title/Abstract]
  OR "sudden neonatal death"[Title/Abstract]
  OR "unexpected neonatal death"[Title/Abstract]
  OR "unexpected death"[Title/Abstract]
  OR "unexplained death"[Title/Abstract]
  OR "found dead"[Title/Abstract]
  OR "fatal collapse"[Title/Abstract]
  OR "cot death"[Title/Abstract]
  OR "sudden death"[Title/Abstract]
  OR SUDI[Title/Abstract]
  OR SUID[Title/Abstract]
  OR SUIDS[Title/Abstract]
  OR SIDS[Title/Abstract]
  OR SUDNIC[Title/Abstract]
)
AND
(
  "Infant"[Mesh]
  OR "Infant, Newborn"[Mesh]
  OR infant*[Title/Abstract]
  OR newborn*[Title/Abstract]
  OR neonat*[Title/Abstract]
  OR baby[Title/Abstract]
  OR babies[Title/Abstract]
  OR child*[Title/Abstract]
  OR pediatric*[Title/Abstract]
  OR paediatric*[Title/Abstract]
)
AND
(
  "Nervous System Neoplasms"[Mesh]
  OR "Brain Neoplasms"[Mesh]
  OR "Central Nervous System Neoplasms"[Mesh]
  OR "Spinal Cord Neoplasms"[Mesh]
  OR "Peripheral Nervous System Neoplasms"[Mesh]
  OR "Head and Neck Neoplasms"[Mesh]
  OR "Eye Neoplasms"[Mesh]
  OR "brain tumor"[Title/Abstract]
  OR "brain tumour"[Title/Abstract]
  OR "intracranial tumor"[Title/Abstract]
  OR "intracranial tumour"[Title/Abstract]
  OR "central nervous system tumor"[Title/Abstract]
  OR "central nervous system tumour"[Title/Abstract]
  OR "spinal tumor"[Title/Abstract]
  OR "spinal tumour"[Title/Abstract]
  OR "head and neck tumor"[Title/Abstract]
  OR "head and neck tumour"[Title/Abstract]
  OR "ocular tumor"[Title/Abstract]
  OR "ocular tumour"[Title/Abstract]
  OR "orbital tumor"[Title/Abstract]
  OR "orbital tumour"[Title/Abstract]
  OR glioma*[Title/Abstract]
  OR astrocytoma*[Title/Abstract]
  OR glioblastoma*[Title/Abstract]
  OR oligodendroglioma*[Title/Abstract]
  OR ependymoma*[Title/Abstract]
  OR medulloblastoma*[Title/Abstract]
  OR ganglioglioma*[Title/Abstract]
  OR neurocytoma*[Title/Abstract]
```

```
  OR meningioma*[Title/Abstract]
  OR schwannoma*[Title/Abstract]
  OR neurofibroma*[Title/Abstract]
  OR hemangiopericytoma*[Title/Abstract]
  OR haemangiopericytoma*[Title/Abstract]
  OR "atypical teratoid rhabdoid tumor"[Title/Abstract]
  OR "atypical teratoid rhabdoid tumour"[Title/Abstract]
  OR "embryonal tumor"[Title/Abstract]
  OR "embryonal tumour"[Title/Abstract]
  OR "choroid plexus tumor"[Title/Abstract]
  OR "choroid plexus tumour"[Title/Abstract]
  OR "choroid plexus papilloma"[Title/Abstract]
  OR "choroid plexus carcinoma"[Title/Abstract]
  OR "pineal tumor"[Title/Abstract]
  OR "pineal tumour"[Title/Abstract]
  OR "pineal teratoma"[Title/Abstract]
  OR craniopharyngioma*[Title/Abstract]
  OR retinoblastoma*[Title/Abstract]
  OR neuroectodermal[Title/Abstract]
  OR PNET[Title/Abstract]
)
AND
(
  "Autopsy"[Mesh]
  OR autops*[Title/Abstract]
  OR postmortem[Title/Abstract]
  OR "post-mortem"[Title/Abstract]
  OR "post mortem"[Title/Abstract]
  OR "postmortem examination"[Title/Abstract]
  OR "post-mortem examination"[Title/Abstract]
  OR "post mortem examination"[Title/Abstract]
  OR necrops*[Title/Abstract]
  OR forensic[Title/Abstract]
  OR medicolegal[Title/Abstract]
  OR "medico-legal"[Title/Abstract]
  OR "death investigation"[Title/Abstract]
  OR virtopsy[Title/Abstract]
)
```

PUBMED-04 | Haematological, lymphoid, histiocytic, bone-marrow, and disseminated neoplasms

**Database**	PubMed/MEDLINE
**Purpose**	Supplementary search for leukaemias, lymphomas, haematopoietic, myeloid, lymphoid, plasma-cell, histiocytic, mast-cell, bone-marrow, disseminated, and metastatic neoplasms.
**Date searched**	July 19, 2026
**Records retrieved**	45 results

Exact search string

```
(
  "Sudden Infant Death"[Mesh]
  OR "Death, Sudden"[Mesh]
  OR "sudden unexpected death in infancy"[Title/Abstract]
  OR "sudden unexpected infant death"[Title/Abstract]
  OR "sudden unexplained death in infancy"[Title/Abstract]
  OR "sudden unexplained infant death"[Title/Abstract]
  OR "sudden infant death"[Title/Abstract]
  OR "unexpected infant death"[Title/Abstract]
  OR "sudden neonatal death"[Title/Abstract]
  OR "unexpected neonatal death"[Title/Abstract]
  OR "unexpected death"[Title/Abstract]
  OR "unexplained death"[Title/Abstract]
  OR "found dead"[Title/Abstract]
  OR "fatal collapse"[Title/Abstract]
  OR "cot death"[Title/Abstract]
  OR "sudden death"[Title/Abstract]
  OR SUDI[Title/Abstract]
  OR SUID[Title/Abstract]
  OR SUIDS[Title/Abstract]
  OR SIDS[Title/Abstract]
  OR SUDNIC[Title/Abstract]
)
AND
(
  "Infant"[Mesh]
  OR "Infant, Newborn"[Mesh]
  OR infant*[Title/Abstract]
  OR newborn*[Title/Abstract]
  OR neonat*[Title/Abstract]
  OR baby[Title/Abstract]
  OR babies[Title/Abstract]
  OR child*[Title/Abstract]
  OR pediatric*[Title/Abstract]
  OR paediatric*[Title/Abstract]
)
AND
(
  "Hematologic Neoplasms"[Mesh]
  OR "Leukemia"[Mesh]
  OR "Lymphoma"[Mesh]
  OR "Histiocytosis"[Mesh]
  OR hematologic*[Title/Abstract]
  OR haematologic*[Title/Abstract]
  OR hematopoietic[Title/Abstract]
  OR haematopoietic[Title/Abstract]
  OR leukem*[Title/Abstract]
  OR leukaem*[Title/Abstract]
  OR lymphoma*[Title/Abstract]
  OR lymphoproliferative[Title/Abstract]
  OR myeloproliferative[Title/Abstract]
  OR myelodysplastic[Title/Abstract]
  OR myeloma*[Title/Abstract]
  OR plasmacytoma*[Title/Abstract]
  OR "lymphoblastic lymphoma"[Title/Abstract]
  OR "acute lymphoblastic leukemia"[Title/Abstract]
```

```
    OR "acute lymphoblastic leukaemia"[Title/Abstract]
    OR "acute myeloid leukemia"[Title/Abstract]
    OR "acute myeloid leukaemia"[Title/Abstract]
    OR "acute myelogenous leukemia"[Title/Abstract]
    OR "acute myelogenous leukaemia"[Title/Abstract]
    OR "congenital leukemia"[Title/Abstract]
    OR "congenital leukaemia"[Title/Abstract]
    OR "juvenile myelomonocytic leukemia"[Title/Abstract]
    OR "juvenile myelomonocytic leukaemia"[Title/Abstract]
    OR histiocyt*[Title/Abstract]
    OR "Langerhans cell"[Title/Abstract]
    OR mastocyt*[Title/Abstract]
    OR "bone marrow neoplasm"[Title/Abstract]
    OR "bone marrow tumor"[Title/Abstract]
    OR "bone marrow tumour"[Title/Abstract]
    OR "disseminated neoplasm"[Title/Abstract]
    OR "disseminated tumor"[Title/Abstract]
    OR "disseminated tumour"[Title/Abstract]
    OR "metastatic tumor"[Title/Abstract]
    OR "metastatic tumour"[Title/Abstract]
    OR "metastatic neoplasm"[Title/Abstract]
  )
  AND
  (
    "Autopsy"[Mesh]
    OR autops*[Title/Abstract]
    OR postmortem[Title/Abstract]
    OR "post-mortem"[Title/Abstract]
    OR "post mortem"[Title/Abstract]
    OR "postmortem examination"[Title/Abstract]
    OR "post-mortem examination"[Title/Abstract]
    OR "post mortem examination"[Title/Abstract]
    OR necrops*[Title/Abstract]
    OR forensic[Title/Abstract]
    OR medicolegal[Title/Abstract]
    OR "medico-legal"[Title/Abstract]
    OR "death investigation"[Title/Abstract]
    OR virtopsy[Title/Abstract]
  )
```

PUBMED-05 | Visceral, abdominopelvic, endocrine, germ-cell, soft-tissue, vascular, bone, and cutaneous neoplasms

**Database**	PubMed/MEDLINE
**Purpose**	Supplementary search covering hepatic, renal, adrenal, pancreatic, gastrointestinal, retroperitoneal, pelvic, endocrine, germ-cell, embryonal, soft-tissue, muscle, vascular, bone, splenic, and cutaneous neoplasms.
**Date searched**	July 19, 2026
**Records retrieved**	53 results

Exact search string

```
  (
    "Sudden Infant Death"[Mesh]
    OR "Death, Sudden"[Mesh]
    OR "sudden unexpected death in infancy"[Title/Abstract]
    OR "sudden unexpected infant death"[Title/Abstract]
    OR "sudden unexplained death in infancy"[Title/Abstract]
    OR "sudden unexplained infant death"[Title/Abstract]
    OR "sudden infant death"[Title/Abstract]
    OR "unexpected infant death"[Title/Abstract]
    OR "sudden neonatal death"[Title/Abstract]
    OR "unexpected neonatal death"[Title/Abstract]
    OR "unexpected death"[Title/Abstract]
    OR "unexplained death"[Title/Abstract]
    OR "found dead"[Title/Abstract]
    OR "fatal collapse"[Title/Abstract]
    OR "cot death"[Title/Abstract]
    OR "sudden death"[Title/Abstract]
    OR SUDI[Title/Abstract]
    OR SUID[Title/Abstract]
    OR SUIDS[Title/Abstract]
    OR SIDS[Title/Abstract]
    OR SUDNIC[Title/Abstract]
  )
  AND
  (
    "Infant"[Mesh]
    OR "Infant, Newborn"[Mesh]
    OR infant*[Title/Abstract]
    OR newborn*[Title/Abstract]
    OR neonat*[Title/Abstract]
    OR baby[Title/Abstract]
    OR babies[Title/Abstract]
    OR child*[Title/Abstract]
    OR pediatric*[Title/Abstract]
    OR paediatric*[Title/Abstract]
  )
  AND
  (
    "Neoplasms, Germ Cell and Embryonal"[Mesh]
    OR "Abdominal Neoplasms"[Mesh]
    OR "Digestive System Neoplasms"[Mesh]
    OR "Endocrine Gland Neoplasms"[Mesh]
    OR "Urogenital Neoplasms"[Mesh]
    OR "Pelvic Neoplasms"[Mesh]
    OR "Soft Tissue Neoplasms"[Mesh]
    OR "Muscle Neoplasms"[Mesh]
    OR "Vascular Neoplasms"[Mesh]
    OR "Bone Neoplasms"[Mesh]
    OR "Skin Neoplasms"[Mesh]
    OR "Splenic Neoplasms"[Mesh]
    OR neuroblastoma*[Title/Abstract]
```

```
  OR ganglioneuroblastoma*[Title/Abstract]
  OR ganglioneuroma*[Title/Abstract]
  OR hepatoblastoma*[Title/Abstract]
  OR "liver tumor"[Title/Abstract]
  OR "liver tumour"[Title/Abstract]
  OR "hepatic tumor"[Title/Abstract]
  OR "hepatic tumour"[Title/Abstract]
  OR "hepatic hemangioma"[Title/Abstract]
  OR "hepatic haemangioma"[Title/Abstract]
  OR "infantile hepatic hemangioma"[Title/Abstract]
  OR "infantile hepatic haemangioma"[Title/Abstract]
  OR hemangioendothelioma*[Title/Abstract]
  OR haemangioendothelioma*[Title/Abstract]
  OR angiosarcoma*[Title/Abstract]
  OR "renal tumor"[Title/Abstract]
  OR "renal tumour"[Title/Abstract]
  OR "kidney tumor"[Title/Abstract]
  OR "kidney tumour"[Title/Abstract]
  OR "mesoblastic nephroma"[Title/Abstract]
  OR nephroblastoma*[Title/Abstract]
  OR "Wilms tumor"[Title/Abstract]
  OR "Wilms tumour"[Title/Abstract]
  OR "adrenal tumor"[Title/Abstract]
  OR "adrenal tumour"[Title/Abstract]
  OR "suprarenal tumor"[Title/Abstract]
  OR "suprarenal tumour"[Title/Abstract]
  OR pheochromocytoma*[Title/Abstract]
  OR phaeochromocytoma*[Title/Abstract]
  OR "pancreatic tumor"[Title/Abstract]
  OR "pancreatic tumour"[Title/Abstract]
  OR pancreatoblastoma*[Title/Abstract]
  OR "gastrointestinal tumor"[Title/Abstract]
  OR "gastrointestinal tumour"[Title/Abstract]
  OR "abdominal tumor"[Title/Abstract]
  OR "abdominal tumour"[Title/Abstract]
  OR "retroperitoneal tumor"[Title/Abstract]
  OR "retroperitoneal tumour"[Title/Abstract]
  OR "pelvic tumor"[Title/Abstract]
  OR "pelvic tumour"[Title/Abstract]
  OR "germ cell tumor"[Title/Abstract]
  OR "germ cell tumour"[Title/Abstract]
  OR germinoma*[Title/Abstract]
  OR teratoma*[Title/Abstract]
  OR "yolk sac tumor"[Title/Abstract]
  OR "yolk sac tumour"[Title/Abstract]
  OR "sacrococcygeal tumor"[Title/Abstract]
  OR "sacrococcygeal tumour"[Title/Abstract]
  OR "gonadal tumor"[Title/Abstract]
  OR "gonadal tumour"[Title/Abstract]
  OR "thyroid tumor"[Title/Abstract]
  OR "thyroid tumour"[Title/Abstract]
  OR "parathyroid tumor"[Title/Abstract]
  OR "parathyroid tumour"[Title/Abstract]
  OR "pituitary tumor"[Title/Abstract]
  OR "pituitary tumour"[Title/Abstract]
  OR "soft tissue tumor"[Title/Abstract]
  OR "soft tissue tumour"[Title/Abstract]
  OR rhabdomyosarcoma*[Title/Abstract]
  OR fibrosarcoma*[Title/Abstract]
  OR "infantile fibrosarcoma"[Title/Abstract]
  OR leiomyoma*[Title/Abstract]
  OR leiomyosarcoma*[Title/Abstract]
  OR lipoma*[Title/Abstract]
  OR liposarcoma*[Title/Abstract]
  OR myofibroma*[Title/Abstract]
  OR myofibromatosis[Title/Abstract]
  OR desmoid*[Title/Abstract]
  OR "rhabdoid tumor"[Title/Abstract]
  OR "rhabdoid tumour"[Title/Abstract]
  OR osteosarcoma*[Title/Abstract]
  OR "Ewing sarcoma"[Title/Abstract]
  OR "bone tumor"[Title/Abstract]
  OR "bone tumour"[Title/Abstract]
  OR "skin tumor"[Title/Abstract]
  OR "skin tumour"[Title/Abstract]
  OR melanoma*[Title/Abstract]
  OR hemangioma*[Title/Abstract]
  OR haemangioma*[Title/Abstract]
  OR lymphangioma*[Title/Abstract]
  OR hamartoma*[Title/Abstract]
)
AND
(
  "Autopsy"[Mesh]
  OR autops*[Title/Abstract]
  OR postmortem[Title/Abstract]
  OR "post-mortem"[Title/Abstract]
  OR "post mortem"[Title/Abstract]
  OR "postmortem examination"[Title/Abstract]
  OR "post-mortem examination"[Title/Abstract]
  OR "post mortem examination"[Title/Abstract]
  OR necrops*[Title/Abstract]
  OR forensic[Title/Abstract]
  OR medicolegal[Title/Abstract]
  OR "medico-legal"[Title/Abstract]
  OR "death investigation"[Title/Abstract]
  OR virtopsy[Title/Abstract]
)
```

Scopus search strategies

The following strategies are formatted for direct use in the Scopus Advanced Search interface using the TITLE-ABS-KEY field code.

SCOPUS-01 | Comprehensive autopsy-focused neoplasm search

**Database**	Scopus
**Purpose**	Primary, site-independent search intended to retrieve any benign, malignant, embryonal, haematological, or tumour-like neoplasm detected during a post-mortem investigation of sudden unexpected death. Generic neoplasm and histological stems were used because Scopus does not provide MeSH explosion.
**Date searched**	July 19, 2026
**Records retrieved**	348 results

Exact search string

```
TITLE-ABS-KEY
(
(
  "sudden unexpected death in infancy"
  OR "sudden unexpected infant death"
  OR "sudden unexplained death in infancy"
  OR "sudden unexplained infant death"
  OR "sudden infant death"
  OR "unexpected infant death"
  OR "sudden neonatal death"
  OR "unexpected neonatal death"
  OR "unexpected death"
  OR "unexplained death"
  OR "found dead"
  OR "fatal collapse"
  OR "cot death"
  OR "sudden death"
  OR SUDI
  OR SUID
  OR SUIDS
  OR SIDS
  OR SUDNIC
)
AND
(
  infant*
  OR newborn*
  OR neonat*
  OR baby
  OR babies
  OR child*
  OR pediatric*
  OR paediatric*
)
AND
(
  neoplasm*
  OR neoplasia*
  OR neoplastic
  OR tumor*
  OR tumour*
  OR cancer*
  OR oncolog*
  OR malignan*
  OR carcinoma*
  OR adenocarcinoma*
  OR sarcoma*
  OR blastoma*
  OR leukem*
  OR leukaem*
  OR lymphoma*
  OR myeloma*
  OR histiocyt*
  OR germinoma*
  OR teratoma*
  OR hamartoma*
  OR myofibroma*
  OR papilloma*
  OR glioma*
  OR melanoma*
)
AND
(
  autops*
  OR postmortem
  OR "post-mortem"
  OR "post mortem"
  OR "postmortem examination"
  OR "post-mortem examination"
  OR "post mortem examination"
  OR necrops*
  OR forensic
  OR medicolegal
  OR "medico-legal"
  OR "death investigation"
  OR virtopsy
)
)
```

SCOPUS-02 | Cardiovascular, mediastinal, pulmonary, pleural, and other thoracic neoplasms

**Database**	Scopus
**Purpose**	Supplementary search for cardiac, pericardial, vascular, mediastinal, thymic, pulmonary, pleural, bronchial, tracheal, and other thoracic neoplasms, including rare benign and malignant histological entities.
**Date searched**	July 19, 2026
**Records retrieved**	87 results

Exact search string

```
TITLE-ABS-KEY
(
(
  "sudden unexpected death in infancy"
  OR "sudden unexpected infant death"
  OR "sudden unexplained death in infancy"
  OR "sudden unexplained infant death"
  OR "sudden infant death"
  OR "unexpected infant death"
  OR "sudden neonatal death"
  OR "unexpected neonatal death"
  OR "unexpected death"
  OR "unexplained death"
  OR "found dead"
  OR "fatal collapse"
  OR "cot death"
  OR "sudden death"
  OR SUDI
  OR SUID
  OR SUIDS
  OR SIDS
  OR SUDNIC
)
AND
(
  infant*
  OR newborn*
  OR neonat*
  OR baby
  OR babies
  OR child*
  OR pediatric*
  OR paediatric*
)
AND
(
  "thoracic neoplasm"
  OR "thoracic tumor"
  OR "thoracic tumour"
  OR "cardiac tumor"
  OR "cardiac tumour"
  OR "cardiac neoplasm"
  OR "heart tumor"
  OR "heart tumour"
  OR "intracardiac tumor"
  OR "intracardiac tumour"
  OR "pericardial tumor"
  OR "pericardial tumour"
  OR "mediastinal mass"
  OR "mediastinal tumor"
  OR "mediastinal tumour"
  OR "thymic tumor"
  OR "thymic tumour"
  OR thymoma*
  OR "pulmonary tumor"
  OR "pulmonary tumour"
  OR "lung tumor"
  OR "lung tumour"
  OR "pleural tumor"
  OR "pleural tumour"
  OR "bronchial tumor"
  OR "bronchial tumour"
  OR "tracheal tumor"
  OR "tracheal tumour"
  OR "pleuropulmonary blastoma"
  OR mesothelioma*
  OR rhabdomyoma*
  OR "cardiac fibroma"
  OR "cardiac myxoma"
  OR fibroelastoma*
  OR angiomyoma*
  OR hemangioma*
  OR haemangioma*
  OR lymphangioma*
  OR "inflammatory myofibroblastic tumor"
  OR "inflammatory myofibroblastic tumour"
  OR "myofibroblastic sarcoma"
  OR "cardiac sarcoma"
  OR "pericardial teratoma"
)
AND
(
  autops*
  OR postmortem
  OR "post-mortem"
  OR "post mortem"
  OR "postmortem examination"
  OR "post-mortem examination"
  OR "post mortem examination"
  OR necrops*
  OR forensic
  OR medicolegal
  OR "medico-legal"
```

```
  OR "death investigation"
  OR virtopsy
)
)
```

SCOPUS-03 | Nervous system, head and neck, ocular, and neuroectodermal neoplasms

**Database**	Scopus
**Purpose**	Supplementary search for central and peripheral nervous system, intracranial, spinal, head and neck, ocular, orbital, pineal, choroid plexus, and neuroectodermal neoplasms.
**Date searched**	July 19, 2026
**Records retrieved**	50 results

Exact search string

```
TITLE-ABS-KEY
(
(
  "sudden unexpected death in infancy"
  OR "sudden unexpected infant death"
  OR "sudden unexplained death in infancy"
  OR "sudden unexplained infant death"
  OR "sudden infant death"
  OR "unexpected infant death"
  OR "sudden neonatal death"
  OR "unexpected neonatal death"
  OR "unexpected death"
  OR "unexplained death"
  OR "found dead"
  OR "fatal collapse"
  OR "cot death"
  OR "sudden death"
  OR SUDI
  OR SUID
  OR SUIDS
  OR SIDS
  OR SUDNIC
)
AND
(
  infant*
  OR newborn*
  OR neonat*
  OR baby
  OR babies
  OR child*
  OR pediatric*
  OR paediatric*
)
AND
(
  "nervous system neoplasm"
  OR "brain tumor"
  OR "brain tumour"
  OR "intracranial tumor"
  OR "intracranial tumour"
  OR "central nervous system tumor"
  OR "central nervous system tumour"
  OR "spinal tumor"
  OR "spinal tumour"
  OR "peripheral nerve tumor"
  OR "peripheral nerve tumour"
  OR "head and neck tumor"
  OR "head and neck tumour"
  OR "ocular tumor"
  OR "ocular tumour"
  OR "orbital tumor"
  OR "orbital tumour"
  OR glioma*
  OR astrocytoma*
  OR glioblastoma*
  OR oligodendroglioma*
  OR ependymoma*
  OR medulloblastoma*
  OR ganglioglioma*
  OR neurocytoma*
  OR meningioma*
  OR schwannoma*
  OR neurofibroma*
  OR hemangiopericytoma*
  OR haemangiopericytoma*
  OR "atypical teratoid rhabdoid tumor"
  OR "atypical teratoid rhabdoid tumour"
  OR "embryonal tumor"
  OR "embryonal tumour"
  OR "choroid plexus tumor"
  OR "choroid plexus tumour"
  OR "choroid plexus papilloma"
  OR "choroid plexus carcinoma"
  OR "pineal tumor"
  OR "pineal tumour"
  OR "pineal teratoma"
  OR craniopharyngioma*
  OR retinoblastoma*
  OR neuroectodermal
  OR PNET
)
AND
(
  autops*
  OR postmortem
  OR "post-mortem"
```

```
  OR "post mortem"
  OR "postmortem examination"
  OR "post-mortem examination"
  OR "post mortem examination"
  OR necrops*
  OR forensic
  OR medicolegal
  OR "medico-legal"
  OR "death investigation"
  OR virtopsy
)
)
```

SCOPUS-04 | Haematological, lymphoid, histiocytic, bone-marrow, and disseminated neoplasms

**Database**	Scopus
**Purpose**	Supplementary search for leukaemias, lymphomas, haematopoietic, myeloid, lymphoid, plasma-cell, histiocytic, mast-cell, bone-marrow, disseminated, and metastatic neoplasms.
**Date searched**	July 19, 2026
**Records retrieved**	81 results

Exact search string

```
TITLE-ABS-KEY
(
(
  "sudden unexpected death in infancy"
  OR "sudden unexpected infant death"
  OR "sudden unexplained death in infancy"
  OR "sudden unexplained infant death"
  OR "sudden infant death"
  OR "unexpected infant death"
  OR "sudden neonatal death"
  OR "unexpected neonatal death"
  OR "unexpected death"
  OR "unexplained death"
  OR "found dead"
  OR "fatal collapse"
  OR "cot death"
  OR "sudden death"
  OR SUDI
  OR SUID
  OR SUIDS
  OR SIDS
  OR SUDNIC
)
AND
(
  infant*
  OR newborn*
  OR neonat*
  OR baby
  OR babies
  OR child*
  OR pediatric*
  OR paediatric*
)
AND
(
  "hematologic neoplasm"
  OR "haematological neoplasm"
  OR hematopoietic
  OR haematopoietic
  OR leukem*
  OR leukaem*
  OR lymphoma*
  OR lymphoproliferative
  OR myeloproliferative
  OR myelodysplastic
  OR myeloma*
  OR plasmacytoma*
  OR "lymphoblastic lymphoma"
  OR "acute lymphoblastic leukemia"
  OR "acute lymphoblastic leukaemia"
  OR "acute myeloid leukemia"
  OR "acute myeloid leukaemia"
  OR "acute myelogenous leukemia"
  OR "acute myelogenous leukaemia"
  OR "congenital leukemia"
  OR "congenital leukaemia"
  OR "juvenile myelomonocytic leukemia"
  OR "juvenile myelomonocytic leukaemia"
  OR histiocyt*
  OR "Langerhans cell"
  OR mastocyt*
  OR "bone marrow neoplasm"
  OR "bone marrow tumor"
  OR "bone marrow tumour"
  OR "disseminated neoplasm"
  OR "disseminated tumor"
  OR "disseminated tumour"
  OR "metastatic tumor"
  OR "metastatic tumour"
  OR "metastatic neoplasm"
)
AND
(
  autops*
  OR postmortem
  OR "post-mortem"
  OR "post mortem"
  OR "postmortem examination"
  OR "post-mortem examination"
  OR "post mortem examination"
  OR necrops*
```

```
  OR forensic
  OR medicolegal
  OR "medico-legal"
  OR "death investigation"
  OR virtopsy
)
)
```

SCOPUS-05 | Visceral, abdominopelvic, endocrine, germ-cell, soft-tissue, vascular, bone, and cutaneous neoplasms

**Database**	Scopus
**Purpose**	Supplementary search covering hepatic, renal, adrenal, pancreatic, gastrointestinal, retroperitoneal, pelvic, endocrine, germ-cell, embryonal, soft-tissue, muscle, vascular, bone, splenic, and cutaneous neoplasms.
**Date searched**	July 19, 2026
**Records retrieved**	79 results

Exact search string

```
TITLE-ABS-KEY
(
(
  "sudden unexpected death in infancy"
  OR "sudden unexpected infant death"
  OR "sudden unexplained death in infancy"
  OR "sudden unexplained infant death"
  OR "sudden infant death"
  OR "unexpected infant death"
  OR "sudden neonatal death"
  OR "unexpected neonatal death"
  OR "unexpected death"
  OR "unexplained death"
  OR "found dead"
  OR "fatal collapse"
  OR "cot death"
  OR "sudden death"
  OR SUDI
  OR SUID
  OR SUIDS
  OR SIDS
  OR SUDNIC
)
AND
(
  infant*
  OR newborn*
  OR neonat*
  OR baby
  OR babies
  OR child*
  OR pediatric*
  OR paediatric*
)
AND
(
  neuroblastoma*
  OR ganglioneuroblastoma*
  OR ganglioneuroma*
  OR hepatoblastoma*
  OR "liver tumor"
  OR "liver tumour"
  OR "hepatic tumor"
  OR "hepatic tumour"
  OR "hepatic hemangioma"
  OR "hepatic haemangioma"
  OR "infantile hepatic hemangioma"
  OR "infantile hepatic haemangioma"
  OR hemangioendothelioma*
  OR haemangioendothelioma*
  OR angiosarcoma*
  OR "renal tumor"
  OR "renal tumour"
  OR "kidney tumor"
  OR "kidney tumour"
  OR "mesoblastic nephroma"
  OR nephroblastoma*
  OR "Wilms tumor"
  OR "Wilms tumour"
  OR "adrenal tumor"
  OR "adrenal tumour"
  OR "suprarenal tumor"
  OR "suprarenal tumour"
  OR pheochromocytoma*
  OR phaeochromocytoma*
  OR "pancreatic tumor"
  OR "pancreatic tumour"
  OR pancreatoblastoma*
  OR "gastrointestinal tumor"
  OR "gastrointestinal tumour"
  OR "abdominal tumor"
  OR "abdominal tumour"
  OR "retroperitoneal tumor"
  OR "retroperitoneal tumour"
  OR "pelvic tumor"
  OR "pelvic tumour"
  OR "germ cell tumor"
  OR "germ cell tumour"
  OR germinoma*
  OR teratoma*
  OR "yolk sac tumor"
  OR "yolk sac tumour"
  OR "sacrococcygeal tumor"
```

```
  OR "sacrococcygeal tumour"
  OR "gonadal tumor"
  OR "gonadal tumour"
  OR "thyroid tumor"
  OR "thyroid tumour"
  OR "parathyroid tumor"
  OR "parathyroid tumour"
  OR "pituitary tumor"
  OR "pituitary tumour"
  OR "soft tissue tumor"
  OR "soft tissue tumour"
  OR rhabdomyosarcoma*
  OR fibrosarcoma*
  OR "infantile fibrosarcoma"
  OR leiomyoma*
  OR leiomyosarcoma*
  OR lipoma*
  OR liposarcoma*
  OR myofibroma*
  OR myofibromatosis
  OR desmoid*
  OR "rhabdoid tumor"
  OR "rhabdoid tumour"
  OR osteosarcoma*
  OR "Ewing sarcoma"
  OR "bone tumor"
  OR "bone tumour"
  OR "skin tumor"
  OR "skin tumour"
  OR melanoma*
  OR hemangioma*
  OR haemangioma*
  OR lymphangioma*
  OR hamartoma*
  OR "abdominal neoplasm"
  OR "digestive system neoplasm"
  OR "endocrine neoplasm"
  OR "urogenital neoplasm"
  OR "pelvic neoplasm"
  OR "soft tissue neoplasm"
  OR "muscle neoplasm"
  OR "vascular neoplasm"
  OR "bone neoplasm"
  OR "skin neoplasm"
  OR "splenic neoplasm"
  OR "germ cell neoplasm"
  OR "embryonal neoplasm"
)
AND
(
  autops*
  OR postmortem
  OR "post-mortem"
  OR "post mortem"
  OR "postmortem examination"
  OR "post-mortem examination"
  OR "post mortem examination"
  OR necrops*
  OR forensic
  OR medicolegal
  OR "medico-legal"
  OR "death investigation"
  OR virtopsy
)
)
```
